# Ultrafast Postcolumn Microdroplet Derivatization of Nucleobases for Enhanced Online Detection, Characterization, and Quantification of Nucleic Acid Modifications Using LC-MS^2^

**DOI:** 10.1021/acs.analchem.6c00004

**Published:** 2026-05-28

**Authors:** Quynh-Trang Do, Husam Kafeenah, Ching-Hua Huang, Shu-Hui Chen

**Affiliations:** Department of Chemistry, 34912 National Cheng Kung University, No.1 College Road, Tainan 701, Taiwan

## Abstract

Modified DNAs and RNAs often exist in trace amounts, which makes detection challenging. Adding ionizable groups to nucleobases (NBs) can enhance sensitivity in liquid chromatography-tandem mass spectrometry (LC-MS^2^). Traditional derivatization methods for modified nucleosides require complex preparation under harsh conditions and cover only limited modifications. We present an ultrafast (microseconds) derivatization technique using ionizable imidazole, facilitated by microdroplets through supersonic electrospray-induced aldehyde condensation for direct LC-MS^2^ analysis. This technique universally tags the exocyclic amine (−NH_2_) or pyrrolic nitrogen (−NH) of five NBs without base damage, particularly guanosine, which is prone to oxidation. Sensitive modifications located on ring carbon, like oxidative lesions and epigenetic marks, or nontagging sites of ring nitrogen, like N-alkylation on guanosine, and depurinating adducts, remain intact. The rapid derivatization avoids artifact generation and allows online postcolumn *in situ* detection, maintaining chromatographic separation. This method achieves over 90% derivatization ratio and improves quantification sensitivity by more than 10-fold. It also enables a quick test for tagging site-blocked modifications. We demonstrate this method by locating stable adduction sites of 4-hydroxy-estradiol (4OHE2) in chromatin DNA and quantifying 4OHE2-induced oxidation and released adducts in MCF-7 cells, achieving 13–43-fold higher sensitivity with significantly reduced sample amounts. Key parameters influencing detection efficiency were systematically explored for routine application.

## Graphical abstract

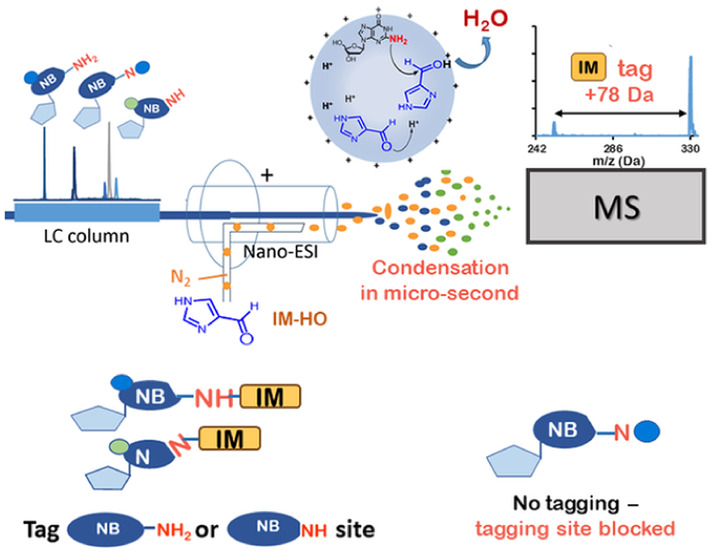

## Introduction

Accurate measurement of various DNA and RNA modifications is an important first step in understanding the biological consequences of these modifications and the potential diseases they may cause.^1–3^ Liquid chromatography-tandem mass spectrometry (LC-MS^2^) is a powerful tool for identifying, characterizing, and quantifying DNA and RNA modifications.^2^ Standard workflows involve enzymatic hydrolysis to nucleosides,^1^ which improves detection sensitivity by reducing molecular size and preventing ion suppression caused by phosphates. However, many modifications occur at extremely low abundance, one per 10^6^ canonical nucleosides,^4^ making them challenging to detect.

Chemical derivatization has been developed to improve MS sensitivity by introducing ionizable tags to modified nucleosides,^5–14^ as summarized in Table 1. In general, available derivatization methods are rare among the over 150 nucleic acid modifications that have been identified.^15^ Most derivatization methods are limited to a subset of modifications, mainly at cytidine (C) or uracil (U), by targeting various sites. For instance, Girard reagents target the modified formyl^5,6^ or carboxyl group^7^ on the C5 of the C/U base, and dansylhydrazine (DNSH) targets the hydroxymethyl group via oxidation to a carbonyl group.^8^ Other reagents target specific nitrogen sites on the C/U base; for example, dimethylamino benzoic anhydride or *N*-dimethylamino-l-naphthalene-1-sulfonyl chloride (Dns-Cl) targets the exocyclic amine of C (NH_2_–C); *N*-cyclohexyl-*N*′-β-(4-methylmorpholinium)­ethylcarbodiimide *p*-toluenesulfonate (CMCT) targets the N3 of U (N3–U), and 2-bromo-1-(4-dimethylamino-phenyl)-ethanone (BDAPE) or 2-bromo-2′-acetonaphthone (BAN) targets the exocyclic amine (NH_2_–C) and N3 of C (N3–C) by forming pentacyclic binding. Additionally, Dns-Cl can tag adenine (A) by targeting its exocyclic amine (NH_2_-A); BPAP or BAN can also tag A by targeting the N9-A or its nucleosides if the β-N9-glycosidic bond is released. In general, derivatization methods targeting the NB sites can be applied to various modifications, as long as these modifications do not block the target sites. However, none is reported for modifications on guanine (G) or its nucleosides, which has the lowest redox potential^16^ among the five NBs. Most conventional methods often require significantly excess reagents and acid/base/metal catalysis or high temperatures, involving hours of reactions (Table 1), which may affect the labile G or sensitive modifications on nucleosides such as oxidative lesions 8-oxo-7,8-dihydro-2′-deoxyguanosine (8oxo-dG), 8-oxo-7,8-dihydro-2′-deoxyadenosine (8oxo-dA) which undergo secondary oxidation,^17,18^ alkylated deoxypurines like N3-methyl-2′-deoxyadenosine (N3me-dA) or N7-methyl-2′-deoxyguanosine (N7me-dG), which depurinate readily, leading to abasic sites in DNA,^19^ pseudouridine (Ψ), which can isomerize or degrade, or epigenetic marks like 5-formyl-2′-deoxycytidine (5f-dC) or 5-carboxyl-2′-deoxycytidine (5ca-dC), which are prone to decarboxylation.^20–22^ Additional cleanup and enrichment processes or special handling steps are carried out during streamlined preparation (Table 1) to concentrate the products while minimizing degradation or chemical artifacts.^9,12^

**Table 1:** Comparability of Derivatization Methods for Detecting Nucleoside Modifications Using LC-MS

Modified nucleosides (examples demonstrated)^a^	Online/Offline	Derivatization reagent^b^	Target site	Reaction conditions (time, temp/molar ratio/catalyst-environment	LOQ^c^ or LOD^c^ (on column)	Fold-enhancement (LOD)	Advantages/Disadvantages	Refs
5f-dU	Offline	Girard T	Formyl modification	10 min, RT/30:1/10% acetic acid	2–4 fmol LOQ	∼20	-fast (10 min), selective for formyl modification	^5^
							-Requires excess reagent, acid catalyst and additional cleanup steps, limited to formyl modifications.	
5f-dC, 5f-U; 5f-rC, 5f-rU, 5f-rCme, 5f-rUme	Offline	Girard T/P/4-APC	Formyl modification	5 min, 30 °C/>50:1/no catalyst	0.1 fmol LOQ	120–880	-Fast (5 min), catalyst-free, SPME cleanup and preconcentration, selective for formyl modification. Sensitivity enhancement >100 fold	^6^
							-Limited to formyl modifications, needs excess reagent and additional cleanup step.	
5f-dC, 5ca-dC	Offline	Girard T/P/D	Formyl and carboxyl modification	5–40 min, 40 °C/150:1/CMPI + TEA	0.03–0.42 fmol LOD	52–260	-Selective for formyl and carboxyl modification.	^7^
							-Limited to formyl or carboxyl modification, excess reagent, harsh catalysts.	
5hm-dC, 5f-dC, 5hm-rC, 5f-rC	Offline	MnO_2_ oxidation + DNSH	Formyl and hydroxymethyl modification	1–3 h, 40 °C/not reported/MnO_2_ + HAc	0.03–0.04 fmol LOQ	363–380	-Selective for formyl and hydroxymethyl modification, improve LC separation, Sensitivity enhancement >100 fold	^8^
							-Limited to formyl or hydroxymethyl modification, requires MnO_2_ preoxidation and additional cleanup	
5me-dC, 5hm-dC, 5f-dC, and 5ca-dC	Offline	BDAPE	pentacyclic binding between N4–C and N3–C	6 h, 60 °C/4000:1/4 mM TEA	0.1–0.23 fmol LOD	35–123	-Broad applicable for C modifications (methylation, oxidation etc.), improve LC separation, Sensitivity enhancement >100 fold	^9^
							-Limited to modified C, long reaction time at high temperature, TEA base catalyst and excess reagent.	
A, N9-me-A, dC, 5me-dC, 5hm-dC	Offline	BPAP/BAN	N9-A (major); pentacyclic binding: NH_2_–C and N3–C	4–11 h/80 °C/>10:1/TEA and mild acidic condition	0.005–0.02 ng/mL LOQ	5	- Broad applicable for C modifications and depurinating A, improve LC separation.	^10^
							-Long reaction time and heating (80 °C for 11 h), TEA catalyst, and excess reagent.	
rU, Ψ, 5me-rU, 6me-rU, 5hm-rU, 5mo-rU, G, Inosine (noncanonical)	Offline	CMCT + CeO_2_ SPE	N3–U or N1-A of inosine	14 h/40 °C/not reported/borate buffer pH 8.5, CeO_2_ to remove excess CMCT	0.3–2.3 fmol LOD	6–1408	Applicable to various modifications of U and inosine, relatively mild reaction condition Sensitivity enhancement up to >1000 fold	^11,14^
							Long reaction time and extra postderivatization processing to cleanup excess reagent	
5me-dC, 5hm-dC, 5f-dC, 5ca-dC	Offline	4-(dimethylamino) benzoic anhydride	NH_2_–C	3 h at 90 °C/20:1/DIPEA and DMAP	1.2–2.5 fmol LOQ	Not reported	Simultaneous labeling all dC derivatives	^12^
							-Relatively harsh reaction condition	
dC, 5me-dC, dA^c^, 6me-dA,^d^ C^c^, 5me-C, A^c^	Offline	Dns-Cl/Dens-Cl	NH_2_-A/C	1 h/30 °C/not reported/Na_2_CO_3_–NaHCO_3_ reaction buffer at pH 11	20 fmol LOQ	20–400	Applicable to not only nucleobase/nucleoside but also amino acid. Internal standard can be prepared by a similar reagent (Dens-Cl)	^13^
							Extreme alkaline pH up to 1 h. Mainly react with amino acid, need additional cleanup process to remove excess reagent	
8oxo-dG, 8oxo-dA, N1me-dG, N2dime-dG, 5me-dC, 5f-dC, N7-CE-G,^d^ N3-CE-A,^d^ N7-CE-dG,^d^ rU	Online	IM-CHO	NH_2_-A/C/G(exocyclic amine) or N3-T/U and N1-G (pyrrolic nitrogen)	<1 ms (online)/120 °C interface/>5:1/microdroplet-accelerated reaction	0.05–28 fmolLOQ	8–46	Mild,catalyst-free, and ultrafast (<1 ms); general approach to derivatize purine and pyrimidine bases, reduces sample need and preparation steps, quick test for modification sites	This work
							Ineffective for modifications blocking the exocyclic amine sites like 4OHE2-dG/dA, HNE-dG/dA, requires optimization of specialized setup	

a Nucleobase (NB)/nucleoside and modification abbreviations. A/G/T/C/U denote NBs of adenine/guanine/thymine/cytosine/uracil. dA/dG/dT/dC/dU stands for deoxyadenosine/deoxyguanosine/deoxythymidine/deoxycytidine/deoxyuridine. rA/rG/rT/rC/rU stands for adenosine/guanosine/thymidine/cytidine/uridine in RNA. Modification abbreviations: formyl (f), carboxyl (ca), methyl (me), hydroxymethyl (hm), methoxyl (mo). Modifications occur on exocyclic amines unless otherwise specified.

b Abbreviation: Girard T/P/D, Girard reagent T/P/D; 4-APC, 4-(2-(trimethylammonio)­ethoxy)­benzenaminium halide; CMPI, 1-chloro-4-methylpyridinium iodide; TEA, trimethylamine; DNSH, dansylhydrazine; BDAPE, 2-bromo-1-(4-dimethylamino-phenyl)-ethanone; BPAP, 2-bromo-4́-phenylacetophenone; BAN, 2-bromo-2′-acetonaphthone; CMCT, *N*-cyclohexyl-*N*′-β-(4-methylmorpholinium)­ethylcarbodiimide *p*-toluenesulfonate; Dns-Cl, *N*-dimethylamino-l-naphthalene-1-sulfonyl chloride; Dens-Cl, *N*-diethyl-amino-*l*-naphthalene-1-sulfonyl chloride.

c Signal-to-noise ratio (S/N) > 3 for the limit of detection (LOD) and S/*N* > 8 for the limit of quantification (LOQ).

d CE (catechol estrogen) covers 4OHE1, 4OHE2, and 4OHEE2.

Ultrafast microdroplet chemistry can dramatically accelerate various slow chemical^23–26^ or biochemical reactions^27,28^ under mild conditions by utilizing the air/solution interface of tiny droplets, thereby offering an attractive alternative for chemical reactions. Unlike bulk-phase reactions, microdroplet reactions are predominantly accelerated at the air–water interface, where a unique catalytic environment is produced by strong interfacial electrostatic potentials and ultrafast solvent evaporation. These confined and highly polar interfaces fundamentally alter chemoselectivity and substrate specificity compared with bulk solutions. Substrates that can be enriched at the droplet surface and establish favorable physical contact with their reaction partners within the ultrashort (microsecond) droplet lifetime^28^ can react efficiently. A representative example is the superacidic microdroplet environment that enables sulfur fluoride exchange (SuFEx) during concerted Aza-Michael and SuFEx reactions: protonation of the fluorine atom generates an excellent leaving group that rapidly evaporates from the droplet surface to drive the reaction rapidly.^29^ Moreover, sterically crowded aromatic amines showed little or no reaction induced during the ultrashort droplet lifetime, in contrast to less hindered analogues.^29^ By similar mechanisms, the superacidic microdroplet environment has been shown to accelerate slow aldol condensation or amine condensation with aldehyde/ketone^30–32^ in which the condensed leaving group quickly evaporates from the droplet surface. Such microdroplet condensation reactions have been applied to derivatize methanal, ethanal, propanal, and *n*-butanal in beer with 2,4-dinitrophenylhydrazine in the ion source, enabling high-throughput screening through a cross-beam setup.^33^ Similarly, free fatty acids extracted from liver and kidney samples were shown to be rapidly derivatized with 2-picolylamine in an electrospray process, achieving low quantification limits.^34^ Based on these studies, we hypothesized that microdroplets would affect the kinetic and thermodynamic properties of NBs, whose ring nitrogens have relatively weak nucleophilicity due to amine-imine tautomerism or the resonance electron delocalization effect,^35,36^ inducing amine condensation reactions under a mild environment, thereby conserving intact structures for the bases and their modifications. We focused on the derivatization of NBs using imidazole-4-carboxaldehyde (IM-CHO), which contains an aldehyde group for amine condensation reactions and a highly ionizable imidazole (IM) moiety for enhanced MS detection.

Here, the microdroplet derivatization was developed for online postcolumn *in situ* detection by MS. The IM-CHO supersonic spray fuses with NB-containing droplets eluted from an LC column, inducing ultrafast condensation reactions within the ion source of electrospray without affecting chromatographic separation. Critical interface parameters affecting the postcolumn derivatization ratio were systematically studied and optimized to fine-tune the efficiency. We applied this method to characterize and quantify DNA damage induced by genotoxic catechol estrogens,^37^ specifically 4-hydroxyestradiol (4OHE2) and its clickable analogue, 4-hydroxyethinylestradiol (4OHEE2), in MCF-7 breast cancer cells. 4OHE2/4OHEE2 is among the most genotoxic estrogen metabolites, and the level of 4OHE2/4OHEE2-protein adducts^38,39^ in human blood or DNA adducts in human urine,^40,41^ serum,^42^ and tissue^43^ has been correlated with a increased risk of breast cancer and other diseases. 4OHE2-induced chromatin damage has been recently mapped genome-wide by Click-Seq.^44^ Detection of the DNA lesions or released adduct markers is, however, still limited by sensitivity. 4OHE2-induced nucleic acid modifications, such as oxidative lesions (e.g., 8oxo-dG and 8oxo-dA) or alkylated purine bases (e.g., N7me-dG), are relatively fragile, and no derivatization methods have been reported for these modifications.

## Experimental Section

### Reagents

All reagents and resources used in this study are listed in the SI materials, including complete supplier information and catalog numbers. Key reagents included IM-CHO (#456128) and 4-dimethylaminopyridine (DMAP) (#8.51055), both from Sigma-Aldrich (St. Louis, MO, USA). The IM-CHO stock solution prepared in DMSO solvent was stored at −80 °C and confirmed to be stable for over 1 month. The IM-CHO spray reagent was prepared from the stock solution by dilution with working solvents before the experiment. Deoxyadenosine (dA) (#TRC-D232155), deoxyguanosine (dG) (#TRC-D242428), deoxycytidine (dC) (#TRC-D232615), 5me-dC (#TRC-M295900), deoxythymidine (dT) (#TRC-T412000), 4-hydroxyestrone-N7-guanine (4OHE1-G) (#TRC-H942120), 4OHEE2 (#TRC-H942005), 4OHE2 (#TRC-H941895), 8oxo-dG (#TRC-O850250), and 8oxo-dA (#TRC-O850225) were purchased from Toronto Research Chemicals (North York, Canada). *N,N*-Dimethylguanosine (N2dime-dG) (#35347) was purchased from Cayman Chemical (Michigan, USA). 2′-Deoxy-N1-methylguanosine (N1me-dG) (#HY-154147) was purchased from MedChemExpress (NJ, USA). IM was purchased from Fluka Chemie GmbH (Buchs, Switzerland). Uridine 5′-monophosphate disodium salt (UMP) (#A18601-22) was from Thermo Fisher Scientific Inc. (Waltham, MA, USA).

### Cell Culture

Human breast cancer cells (MCF-7) were obtained from the Bioresource Collection and Research Center (Hsinchu, Taiwan). Cells were cultured in 15 cm dishes in DMEM supplemented with 10% FBS and maintained in a humidified incubator at 37 °C with 5% CO_2_.

### Preparation of Standards

Individual stock solutions were prepared in ACN at 1 mg/mL and stored at −80 °C until use. A standard mixture was freshly prepared by mixing an appropriate amount of individual stock solutions (dA, dG, dC, 5me-dC, dT, 8oxo-dG, 8oxo-dA, N2dime-dG, N1me-dG, and 4OHE1-G) and diluting it with H_2_O to yield a final concentration ranging from 5 pg/mL to 10 μg/mL. Calibration standards were generated through serial dilution of the working mixture.

To prepare Uridine (rU), UMP (0.31 mM) was dissolved in Tris–HCl buffer (50 mM, pH 8.5) supplemented with 1 mM MgCl_2_, followed by the addition of alkaline phosphatase (5 U/mL). The reaction was incubated at 37 °C for 5 h, then dried and reconstituted in 10% ACN for LC-MS^2^ analysis.

### Synthesis of 4OHEE2/4OHE2-Induced Adduct Standards

Adduct standards of 4OHEE2 or 4OHE2 were synthesized following the procedure of Zahid et al.^37^ Briefly, 52 μL of 4OHEE2/4OHE2 (1 μg/μL in ACN/DMSO 80:20) was incubated with activated MnO_2_ (1 mg) for 30 min at −30 °C. The resulting yellowish solution was filtered through a 0.45 μm hydrophilic filter, and ACN was evaporated under nitrogen flow. The quinone (in DMSO) was then reacted with dG or dA (40 μL, 10 μg/μL) at 37 °C for 5 h in 50:50 acetic acid/water to generate depurinating N7-4OHEE2/4OHE2-G and N3-4OHEE2/4OHE2-A adducts, as confirmed by MS1 and MS2 (Figure S1). Concentrations were determined by the calibration curve of N7-4OHE1-G, assuming similar ionization efficiencies. Serial dilutions were then carried out to construct individual calibration curves. All the adduct standards were stored at −20 °C until use. Stable deoxypurine adducts, such as 4OHE2/4OHEE2-deoxyguanosine (4OHE2/4OHEE2-dG), were formed at early reaction stages but degraded upon prolonged incubation at 37 °C;^45^ thus, they were used only for qualitative or semiquantitative analysis.

For IR measurement, MnO_2_-activated 4OHEE2 (200 μg) was reacted with dA (1.6 mg) in 50% acetic acid (v/v) at 37 °C for 5 h in a total volume of 160 μL. The reaction mixture was separated using an HPLC-UV system (ECOM ECP2010 HPLC Pump, Thermo Dionex Ultimate 3000 VWD) equipped with a C18 column (150 × 2.1 mm, 5 μm, 300 Å, ZORBAX), controlled by Chromeleon software (Thermo Scientific). The column was maintained at 50 °C. The mobile phases were water (A) and ACN (B). The gradient was as follows: 20% B (0–2 min), ramp to 50% B (2–10 min), hold for 0.5 min, return to 20% B (10.5–11 min), and reequilibrate for 15 min. Fractions containing 4OHEE2-dA were identified by UV absorbance (260 nm), confirmed by MS, vacuum-dried, and reconstituted in ACN for ATR-FTIR analysis (Bruker Optics Ltd., Coventry, UK).

### Chromatin Isolation and Hydrolysis

Chromatin from MCF-7 cells (1 × 10^7^) was isolated following a modified MNase digestion protocol (SI methods). Briefly, cells were cross-linked with 1% paraformaldehyde, quenched with glycine, permeabilized, and digested with micrococcal nuclease (MNase) to release chromatin. The extracted chromatin (∼500 μg protein and ∼100 μg DNA) was incubated with MnO_2_-activated 4OHEE2 quinone (300 μM, 37 °C for 24 h) to generate 4OHEE2-induced DNA adducts. Depurinating 4OHEE2-A/G adducts were extracted from the supernatant using ACN salting-out extraction,^46^ dried, and reconstituted for Micro-LC-microdroplet derivatization. Stable 4OHEE2-dA/dG adducts in the pellet were recovered by Proteinase K digestion, phenol–chloroform DNA extraction, and enzymatic hydrolysis using a tetra-enzyme mixture.^47^ 4OHEE2-dA/dG adducts were enriched by ACN salting-out extraction,^46^ dried, resuspended in 30 μL of H_2_O/ACN (80:20), and stored at −20 °C until use, and 5 μL was injected for analysis. A complete protocol, including buffer compositions, incubation conditions, enzyme amounts, and extraction steps, is provided in the SI methods.

The recovery of sample extraction following the procedures described above was estimated to be >91%.

### Cell Treatment and Medium Collection

MCF-7 cells were treated with 4OHE2 using a repeated-dose schedule (detailed protocol in SI Methods). Briefly, cells at ∼80% confluence were exposed to 1 μM 4OHE2 for 24 h per cycle. After each treatment, cells were replated and allowed to recover to ∼80% confluence before the next exposure. Three sequential 1 μM treatments were performed, followed by a final treatment with 30 μM 4OHE2. Culture media were collected at the first (24 h), third (168 h), and fifth (312 h) postplating time points and supplemented with 2 mM ascorbic acid. Media from DMSO-treated cells served as a control. Collected culture media were extracted on Sep-Pak C18 cartridges, eluted with organic solvent, dried, and enriched for depurinating DNA adducts via ACN salting-out extraction. The sample was then reconstituted in 20% ACN, and finally, 5 μL was injected for Micro-LC-microdroplet derivatization. All media collections were performed in 3 replicates. The workflow for quantifying total nonmodified nucleobases is detailed in the SI Methods. The recovery of sample extraction following the procedures described above was estimated to be 105%.

### Online Micro-LC-Microdroplet Derivatization-MS^2^

Samples (5 μL) were loaded onto a trap capillary (5 mm × 0.2 mm) packed with C18 reversed-phase (5 μm, VYDAC, Hesperia) and then injected into a separation capillary column (220 mm × 0.2 mm, 1.7 μm, BEH, Waters) using a nano-UPLC system (ACQUITY UPLC, Waters Corporation, Milford, MA, USA). As depicted in Figure 3A, the column outlet was online-coupled to an MS (4000QTRAP, MDS Sciex, Toronto, Canada) through a 30 cm tubing (75 μm ID and 366 μm OD), and a 5 cm spray emitter (30 μm ID and 366 μm OD) mounted on a Universal NanoFlow sprayer stand (Waters). This configuration enabled postcolumn microdroplet derivatization with IM-CHO.

IM-CHO microdroplets were generated using N_2_ nebulizing gas and a tee-valve mixer. IM-CHO solution (2 μg/mL) was delivered at 0.2 μL/min by a syringe pump into one side of the tee, while N_2_ gas (20 psi) was applied to the other side to nebulize the IM-CHO and direct the mist toward the electrospray tip.

Mobile phase A containing 0.1% FA in water, and mobile phase B containing 0.1% FA in ACN. Samples were loaded onto the trap column at 2 μL/min with 1% B for 2 min, followed by a 20 min separation gradient: 10% B for 2 min, 10–30% B in 1 min, 30–70% B over 10 min, held at 70% B for 2 min, and reduced to 10% B in 2 min, held for 3 min at 1.7 μL/min. The system was equilibrated for 10 min between injections.

The MS1, MS2, and MRM data were acquired in positive mode with a 4.5 kV spray voltage using nitrogen as the cone and collision gas. Quantification was based on the peak areas of the precursor/product ion pairs. Instrument control and data analysis were performed using Analyst (ver. 1.6.2). All MRM transitions are listed in SI Table S1. For quantification, as previously used,^44^ 4OHE1-G (10 ppb) was spiked into the sample as an internal standard (IS) to correct for the matrix effect.

### Statistical Analysis

Statistical analyses were performed using Excel. All experiments were performed with biological replicates (*n* = 3). Data were reported as mean ± standard deviation (SD). No data points were excluded. Statistical analysis was conducted by a two-tailed unpaired Student’s *t*-test, *P < 0.05, **P < 0.01.

## Results and Discussion

### Microdroplet NB Derivatization by IM-CHO

To test whether microdroplets can induce the NB derivatization, an individual mixture of IM-CHO with each nucleoside standard—deoxyadenosine (dA), deoxyguanosine (dG), deoxycytidine (dC), deoxythymidine (dT), or uridine (rU)—was directly infused into MS through supersonic electrospray. As shown in Figure 1A, all five canonical NBs were rapidly derivatized, resulting in products with a mass increase of +78 (+IM-CHO–H_2_O). The MS2 spectra for these products displayed three major signals corresponding to the intact derivatized nucleosides, nucleosides after loss of the IM tag (intact-IM), and NBs after glycosidic bond cleavage (intact-IM–glycan) under collision-induced dissociation (see Figure S1 for MS2 spectra). The MS1 and MS2 results indicate that an ultrafast condensation reaction involving water loss (−H_2_O) was triggered by supersonic electrospray—an event not observed in bulk solution even after an overnight incubation with acid catalyst (see Figure S2). Furthermore, no reaction occurred with dG (Figure 1B) when IM without an aldehyde moiety was used as the derivatization reagent, suggesting that NBs were tagged by IM-CHO through an aldehyde–amine condensation reaction.

**Figure 1:**
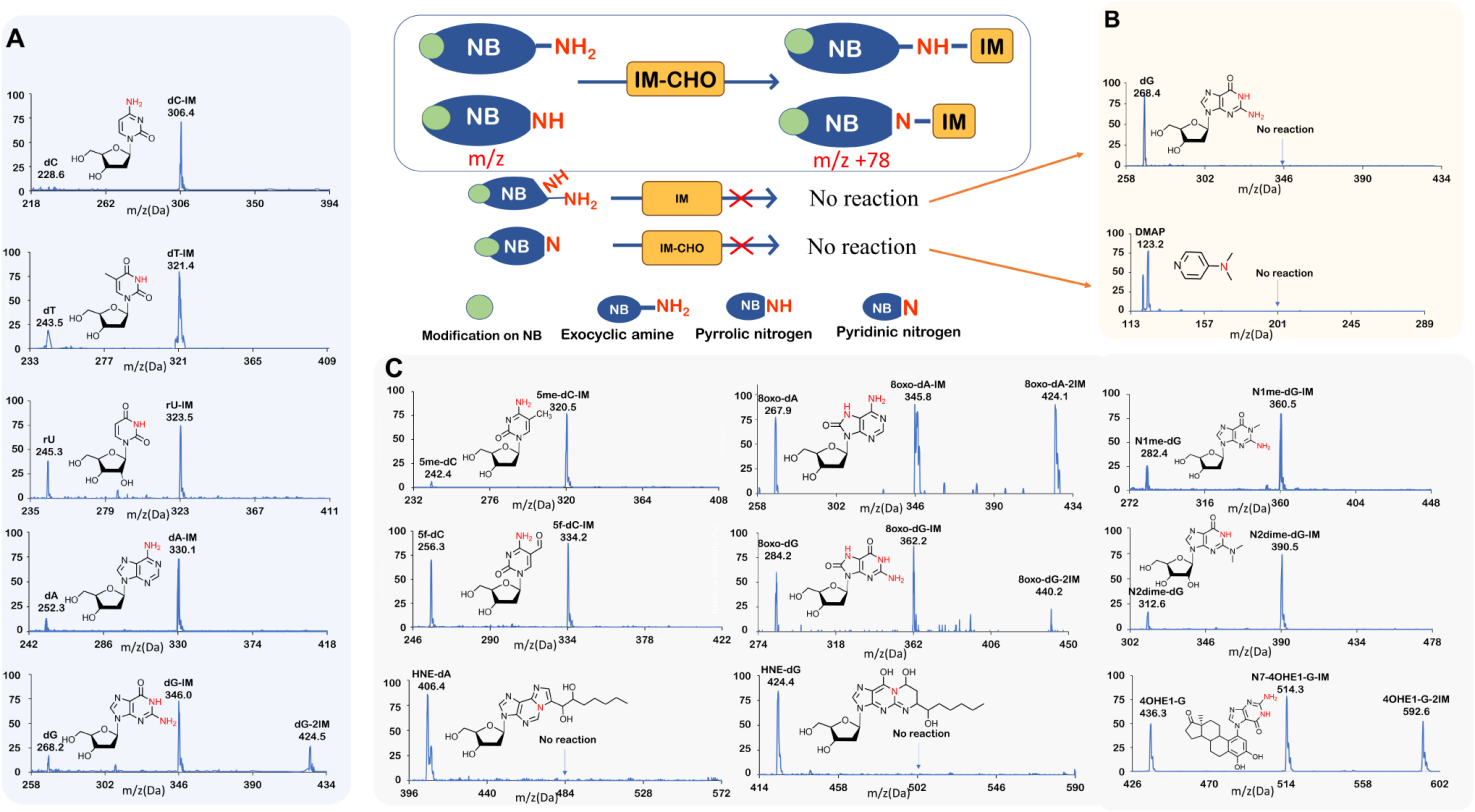
Microdroplet-induced IM-CHO derivatization of nucleosides through aldehyde–amine condensation reaction at exocyclic amines or pyrrolic nitrogens of nucleobases (NBs). MS spectra of *in situ* microdroplet-induced reaction of (A) IM-CHO with the five nucleosides (dC, dT, rU, dA, dG) each yield +78 Da products; (B) IM with dG (top) or IM-CHO with DMAP (bottom), showing no reaction; (C) IM-CHO with nucleosides carrying epigenetic modifications (5me-dC, 5f-dC) and oxidative lesions (8oxo-dG/dA) on the ring carbon, or methylation on the ring nitrogen (N1me-dG, N2dime-dG), showing +78 Da products; IM-CHO with nucleosides carrying cyclic adducts (HNE-dA/dG) that block exocyclic amines and pyrrolic nitrogens, showing no reaction; or IM-CHO with depurinating adducts (N7-4OHE1-G) which retain both tagging sites, produced both +78 Da and +78 × 2 Da products.

### Tagging Sites on NBs of Modified Nucleosides

Among the five NBs, dG, which contains one exocyclic amine (NH_2_-dG) and one pyrrolic nitrogen (NH) on N1-dG, displayed one (+78 × 1) and two (+78 × 2) water-condensed products. In contrast, dA and dC, each having one exocyclic amine (NH_2_-dA/dC), along with dT and rU, which contain one pyrrolic nitrogen (NH) on N3-dT/rU, were detected with only one (+78 × 1) water-condensed product. Additionally, we tested 4-dimethylaminopyridine (DMAP), which has two pyridinic nitrogens (N), and found that it did not induce any detectable signal from the water-condensation reaction (Figure 1B). These findings indicate that the exocyclic amine (−NH_2_) or pyrrolic nitrogen (−NH) of NBs are the main target sites for IM-CHO, leading to aldehyde–amine condensation. Thus, the microdroplet derivatization method using IM-CHO is broadly applicable to modified nucleic acids as long as the modifications do not completely obstruct the tagging sites on the NBs.

To further test this hypothesis, we examined several nucleic acid modifications (Figure 1C). Modifications on ring carbons, including epigenetic marks 5me-dC and 5f-dC, as well as labile oxidative lesions like 8oxo-dG and 8oxo-dA, yielded one or two water-condensed products (top left of Figure 1C). Compared to several published conventional derivatization methods for 5me-dC or 5f-dC (Table 1), our method considerably shortens the derivatization time by 4–5 orders of magnitude. In the case of modifications on the ring nitrogen of dG, N1me-dG and N2dime-dG—both of which have one blocked tagging site—yielded only one (+78 × 1) water-condensed product. Conversely, the depurinating adduct of catechol estrogens (N7-4OHE1-G) retained two accessible sites and was detected with both +78 × 1 and +78 × 2 water-condensed products (right, Figure 1C). Furthermore, 2′-deoxy-adenosine/guanosine-HNE (HNE-dA/dG), which modifies the tagging sites of IM-CHO, including the exocyclic amine (NH_2_-dA/dG) and the N1-dG, by forming a cyclic binding, showed no induced reaction (bottom left of Figure 1C). Generally, the number of IM modifications corresponded to the number of available IM-CHO target sites—the exocyclic amine (−NH_2_) or pyrrolic nitrogen (−NH)—on the NB. The only exception was 8oxo-dG, which possesses three tagging sites (N7-dG induced by C8 oxidation); however, only two out of the three were derivatized by IM-CHO. This likely reflects steric hindrance or insufficient reaction time, as stated above for the concerted Aza-Michael/SuFEx microdroplet reaction system. Among 18 amines tested, sterically crowded aromatic amines bearing substituents near the amine group showed no reaction, in contrast to less hindered analogues.^29^ This highlights the importance of an unhindered physical approach during the microsecond droplet lifetime. Thus, similar effects may occur with the 8oxo-dG microdroplet condensation reaction. The crowded local environment around the third tagging site of 8oxo-dG may physically impede its productive contact with IM-CHO, preventing its reaction during the ultrashort droplet lifetime despite being chemically accessible. Additionally, we confirmed that no side reactions or byproducts were produced, demonstrating that all modifications, including delicate 8oxo-dA/dG, remained intact during microdroplet derivatization (see Figure S3).

A mechanism illustrated in Figure 2 is proposed for the IM-CHO microdroplet condensation reaction with NBs. Since the nucleophilicity of ring nitrogens is relatively weak due to amine-imine tautomerism or the resonance electron delocalization effect,^35,36^ nucleophilicity is not the main driving force to induce aldehyde condensation reaction. Following several mechanisms previously described, based on the unique catalytic environment produced by strong interfacial electrostatic potentials and ultrafast solvent evaporation, we propose that the superacidic microdroplet surface protonates the carbonyl group of IM-CHO, thereby enhancing the nucleophilic attack by the amine groups of NBs. The interface also catalyzes proton transfer from the exocyclic amine (−NH_2_) or pyrrolic nitrogen (−NH) of the NB to generate water as a leaving group. Rapid evaporation of water from the droplet surface helps drive imine formation (R–C—NH^+^ or R–C—N^+^ R) and prevents the hydrolysis of the newly formed imine. These confined and highly polar interfaces fundamentally alter chemo-selectivity and substrate specificity compared with bulk solutions. Only substrates that can be enriched at the droplet surface and establish favorable physical contact with their reaction partners will react efficiently. Thus, the exocyclic amine or pyrrolic nitrogen on NBs serves both as a nucleophile and as a proton donor—an ability absent in pyridinic nitrogens, which lack an N–H proton. As expected, a higher derivatization ratio was observed from the droplet solution that contained 0.1% formic acid compared to one without it (see Figure S4). Although pyridinic nitrogens may be protonated under acidic conditions, protonation of pyridinic nitrogen removes its available lone pair for nucleophilic attack, rendering it unreactive toward carbonyl attack under acidic conditions. Moreover, because the droplet lifetime is extremely short (on the order of microseconds),^28^ all kinetic and thermodynamic factors that normally limit reactions in bulk become decisive determinants of microdroplet selectivity. As discussed above, steric hindrance was thus a potential factor that kinetically governs microdroplet selectivity, leading to the lack of derivatization for the third tagging site of 8oxo-dG.

**Figure 2:**
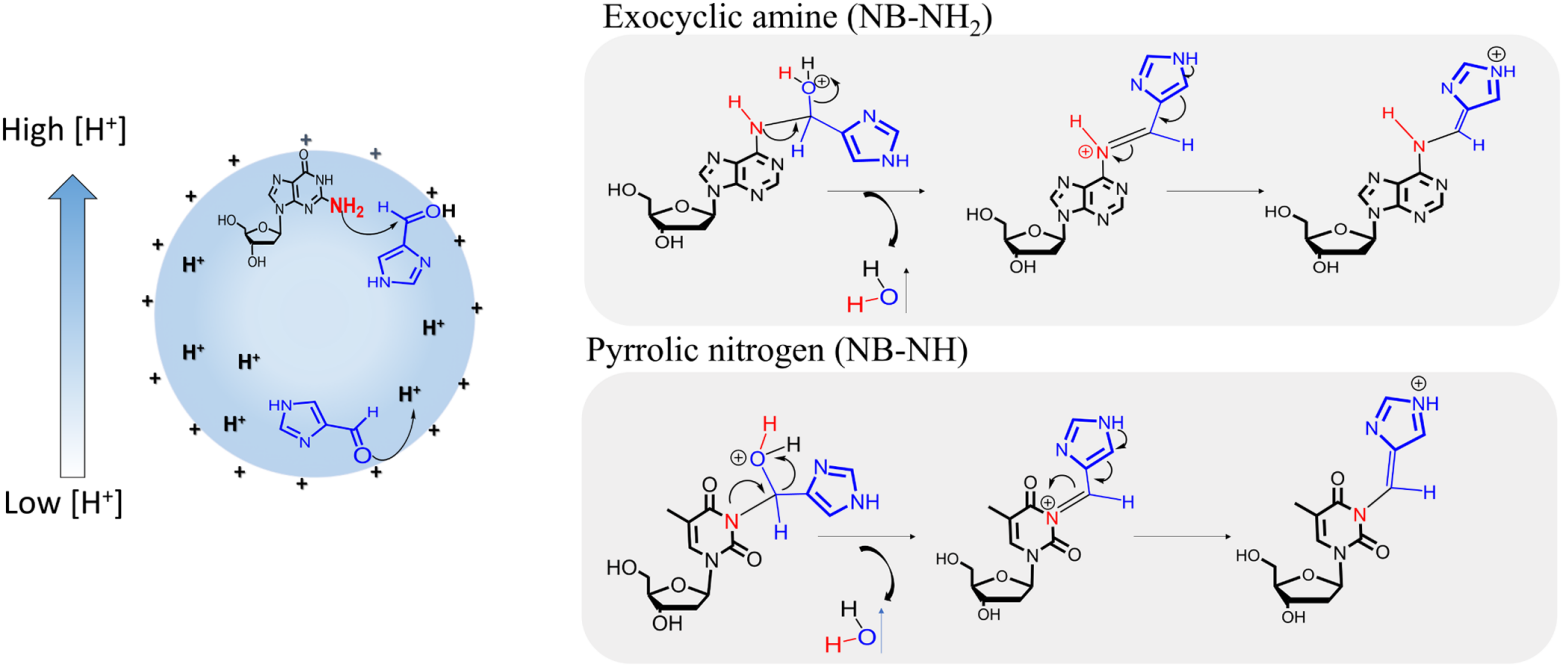
Proposed mechanism of microdroplet-induced IM-CHO derivatization of nucleosides through aldehyde–amine condensation reaction with the exocyclic amine (NB-NH_2_) or pyrrolic nitrogen (NB-NH) of NB.

To test whether microdroplets can promote the nucleophilic attack of an amine group on the carbonyl carbon under neutral conditions through the formation of a carbinolamine intermediate, a common mechanism in bulk solution,^48^ we performed negative-mode electrospray experiments using a neutral mobile phase (pH 6–7) containing ammonium fluoride.^46^ In negatively charged microdroplets, the ring nitrogens of NBs are expected to carry greater electron density, enhancing nucleophilic attack on IM-CHO. Nevertheless, as shown in Figure S5, the intensity of the intact 4OHE1-G [M–H]^−^ ion remained unchanged or slightly increased, with no detectable imine or carbinolamine products. This result further supports the proposed mechanism that the formation and rapid evaporation of water through proton transfer at the superacidic microdroplet interface—not simple nucleophilic attack—is the key driving force for IM-CHO–NB condensation. Importantly, the selectivity patterns observed here align with a dehydration-driven condensation process at the superacidic microdroplet interface and further support the mechanistic interpretation. Experiments with direct structural verification of intermediates within microsecond-lifetime droplets—such as isotopic labeling, trapping of transient intermediates, or comparison with independently synthesized standards—would further strengthen the mechanistic assignment, but these experiments are inherently challenging.

### Optimization of Online Postcolumn IM-CHO Microdroplet Derivatization

To achieve postcolumn online derivatization, as illustrated in Figure 3A, IM-CHO microdroplets generated by N_2_ nebulizing gas were introduced to promote droplet fusion with the LC eluent in electrospray, creating a mist around the electrospray tip. The derivatization products of NBs or nucleosides were analyzed using MS1 and MS2 scans, and the data were employed to establish multiple reaction monitoring (MRM) methods for each NB or modified nucleoside (as shown in Table S1). Various parameters related to the microdroplet fusion configuration required optimization and balance to achieve the highest derivatization efficiency while maintaining optimal ionization efficiency and sensitivity for ESI-MS detection. Because authentic IM-derivatized standards are not currently available, we calculated the derivatization ratio as the peak area of the derivatized species divided by the sum of the derivatized and nonderivatized species to optimize experimental conditions. The derivatization ratio increased with the derivatization yield; however, it does not account for potential differences in ionization efficiencies and, therefore does not represent an absolute chemical yield.

**Figure 3:**
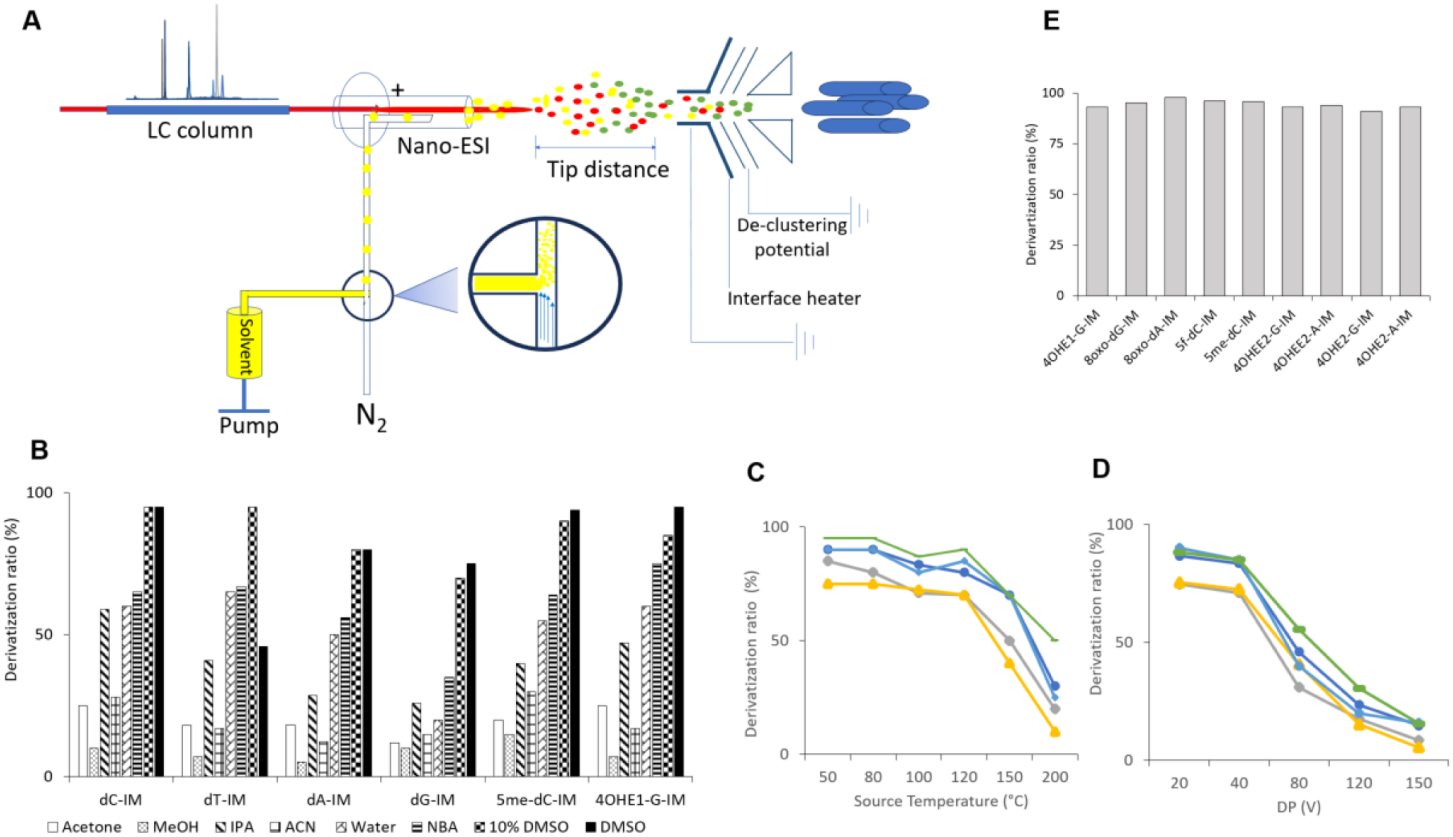
Optimization of online postcolumn microdroplet derivatization. (A) LC-MS setup and derivatization ratio affected by key parameters: (B) IM-CHO reagent solvent (under DP 50, 120 °C), (C) interface temperature (10% DMSO reagent solvent, DP 50), and (D) DP (under 10% DMSO reagent solvent, 120 °C). (E) Overall derivatization ratio of the modified nucleosides under optimized conditions.

The derivatization ratio of IM-CHO was systematically optimized using several tested standards. Optimizations focused on one water-condensed product (78 × 1) from all standards. Among the parameters examined, three were found to significantly impact the derivatization ratio: the solvent composition of the IM-CHO reagent, the interface temperature, and the declustering potential (DP) voltage between the needle tip and the MS orifice.

As shown in Figure 3B, among the seven solvents tested (acetone, methanol, isopropanol, acetonitrile, water, 3-nitrobenzyl alcohol, and DMSO), DMSO produced the highest derivatization ratio for all tested standards (dA, dG, dC, dT, 5me-dC, and N7-4OHE1-G) except for dT. In contrast, acetone and methanol yielded the lowest derivatization ratios across all standards. Generally, the derivatization ratio correlated positively with the boiling point of the solvents: DMSO, with a boiling point of 189 °C, showed the highest yield, while acetone (boiling point 56 °C) and methanol (boiling point 65 °C) showed the lowest. This trend was also consistent with the other four solvents tested: NBA (175–180 °C) > water (100 °C) > IPA (82.3 °C) > ACN (82 °C). Higher derivatization ratios were consistently linked to longer droplet lifetimes, resulting from either higher boiling points or lower volatility of the IM-CHO reagent solvent. The low derivatization ratio for dT (<50%) with the DMSO spray solvent may be due to the nucleophilic nature of DMSO, which could suppress the interaction between IM-CHO and the tagging site of N3-T/U, as it has the weakest nucleophilicity among all the nitrogen in the NBs. This hypothesis was supported by the increased derivatization ratio (>90%) observed when the DMSO concentration was reduced to 10% (Figure 3B). The effect of interface temperature was examined using 10% DMSO as the IM solvent (Figure 3C). Since the binary mixture of water and DMSO does not form an azeotrope, water evaporates from the droplet first at just above 100 °C, followed by the evaporation of DMSO around 189 °C. Consequently, the derivatization ratio for all tested standards was highest (∼90%) at temperatures below 120 °C and gradually decreased to ∼20% at 200 °C. This trend aligns with higher derivatization ratios being associated with longer droplet lifetimes. Additionally, decreasing the DP (Figure 3D), which is utilized to remove solvent clusters before ions enter the mass analyzer, also increased the derivatization ratio, suggesting a longer droplet lifetime with lower DP settings. Additionally, this droplet fusion interface conserved derivatization efficiency while reducing the extracolumn broadening effect induced by using a premix tee for introducing reagents.^49^ Under optimized conditions, the derivatization ratio for modified nucleoside standards (such as N7-4OHE1-G, 8oxo-dG, 8oxo-dA, 5f-dC, and 5me-dC) and 4OHE2/4OHEE2-induced adduct standards (discussed later) exceeded 90% (Figure 3E), leading to >10 times signal enhancement compared to those without derivatization. A limit of quantification (LOQ) of around 10 pg/mL with a signal-to-noise ratio of approximately 8 was obtained by IM-CHO derivatization for N7-4OHE1-G (see Figure S6A).

To evaluate the reproducibility of run-to-run derivatization and potential carryover between injections, three consecutive sample injections were performed, each followed by two blank solvent injections. As shown (SI Figure S7A), the relative standard deviations of the derivatized ion intensities across the three sample injections were 14% for 8oxo-dA-IM and 3% for 4OHE1-G-IM, indicating reproducible online IM-CHO derivatization for quantitative analysis. Moreover, almost no carryover signal (<1%) was detected from the first or the second wash of either 8oxo-dA-IM or 4OHE1-G-IM (SI Figure S7A). These results demonstrate that the carryover is minor (<1%) and does not materially affect quantification. However, it is noticeable that trace amounts of IM-CHO (*m*/*z* = 96) were observed to adsorb to the ion source or quadrupole after extended use (Figure S7B). Although the contaminated IM-CHO could not induce derivatization to affect quantification results if no analyte is injected, it is necessary to clean the instrument with continuous reagent solvent (10% DMSO) spray after a long period of sample runs to minimize adverse effects such as higher background noise. As shown (Figure S7B), the contaminated signal decreased by 90% (10% remaining) after 5 solvent injections and further decreased to 96% after 10 solvent injections. The required instrument cleaning time, however, varies with the running conditions, and justification is required on a case by-case basis . There are potential challenges associated with applying this new online derivatization method to various experimental settings and samples. Nevertheless, since a commercial nanospray interface was used here with minimal modifications, standardized procedures can be established to construct an optimized and reliable method for regular operation on different instruments.

### Application to 4OHE2-Induced DNA Damage in MCF-7 Cells

Using the previously described procedure,^37^ 4OHE2 and 4OHEE2 (clickable analogue) adduct standards were generated through quinone conversion followed by immediate reaction with dA/dG, resulting in depurinating N7-4OHE2/4OHEE2-G or N3-4OHE2/4OHEE2-A and stable 4OHE2/4OHEE2-dA/dG adducts. Similar to N7-4OHE1-G, depurinating N7-4OHE2-G or N3-4OHE2-A (shown at the top of Figure 4A) and their 4OHEE2 adducts (as seen in Figure S8) could be readily derivatized by IM-CHO, achieving over 90% derivatization ratio and more than a 10-fold increase in sensitivity. In contrast, no derivatization occurred with 4OHEE2-dA (middle left of Figure 4A) or 4OHE2-dA (Figure S8). The adduction/modification site of 4OHE2-dA has not been reported and cannot be resolved by MS^2^ (see Figure S1) due to insufficient fragmentation patterns and the lack of authentic standards. The accurate mass of the synthesized 4OHEE2-dA standard (562.1700 experimental value versus 562.2665 theoretical value) and its isotopic pattern (Figure S9) were confirmed by high-resolution MS. The results may thus suggest that the 4OHE2/4OHEE2 modifies the exocyclic amine NH_2_-dA, the only IM-CHO tagging site on dA, leading to no tagging induced by IM-CHO microdroplet derivatization. This assignment was supported by IR spectra (bottom right of Figure 4A), whereby the NH stretching band (∼3250, 3106 cm^–1^) of dA disappeared. However, this modification site remains hypothetical due to weak signal and the lack of fully annotated spectra. For stable 4OHE2/4OHEE2-dG adducts, a low derivatization ratio was observed, accompanied by a weak signal corresponding to one (+78 × 1) water-condensed product (bottom left of Figure 4A and Figure S8), suggesting the presence of minor 4OHE2/4OHEE2 modification/adduction sites which do not block the tagging sites of IM-CHO. Unlike HNE-dG/dA, which forms a cyclic modification that obstructs both IM-CHO targeting sites of dG, 4OHE2/4OHEE2 modification likely blocks one of the two tagging sites while allowing another site to be derivatized by IM-CHO. The MRM methods (Table S1), developed based on these generated 4OHE2/4OHEE2-modified standards along with 8oxo-dA/dG standards, were used to detect, characterize, and quantify damage on DNA and the released adducts of MCF-7 cells caused by 4OHE2/4OHEE2. The chromatin DNA was hydrolyzed to collect nucleosides with the stable and oxidative modifications. The culture medium of 4OHE2/4OHEE2-treated cells was saved to collect the released depurinating adducts.

**Figure 4:**
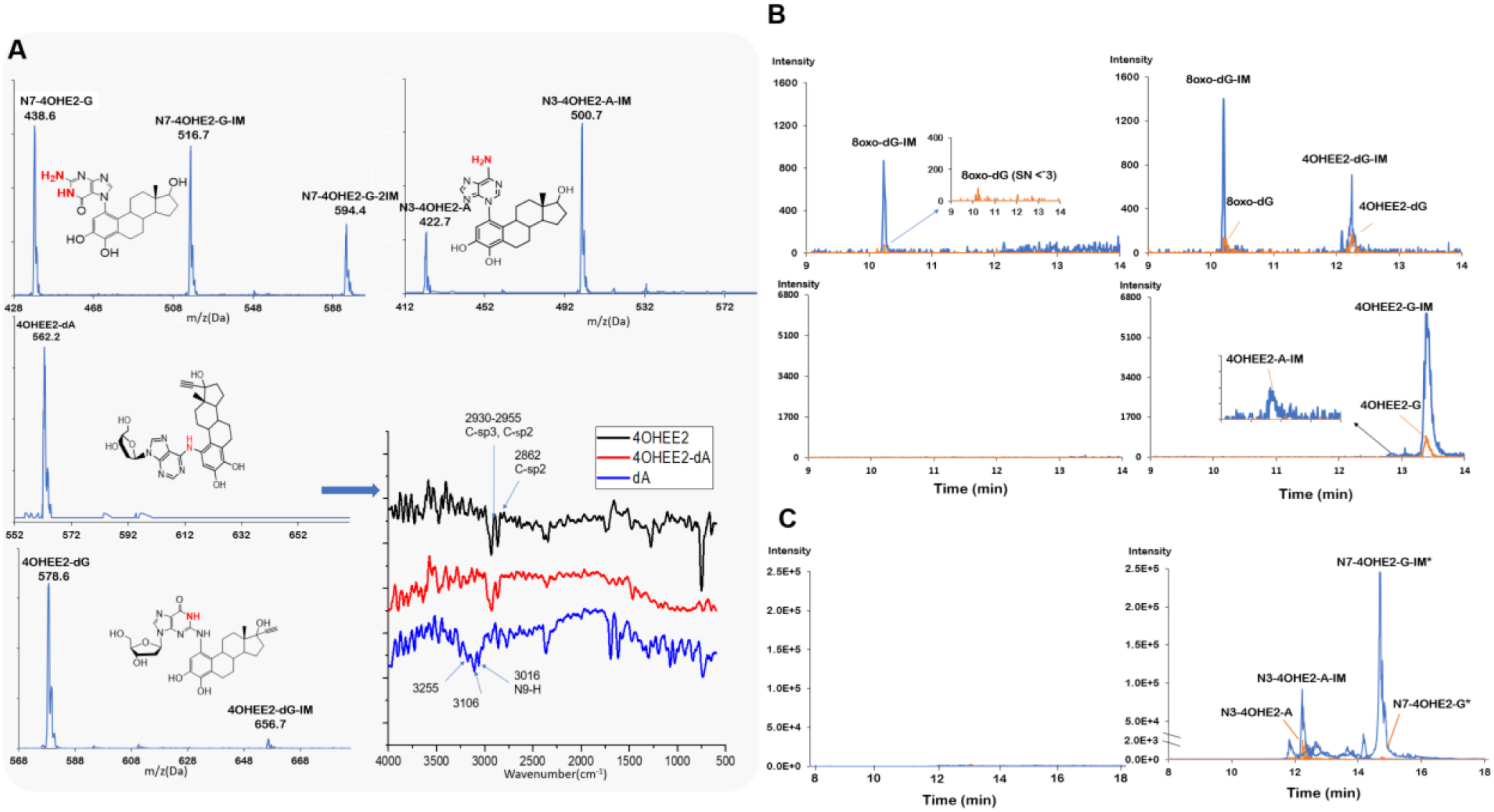
Microdroplet-induced IM-CHO online postcolumn derivatization for detection and characterization of DNA damage caused by 4OHE2/4OHEE2 using LC-MRM. (A) The MS spectra acquired from the 4OHEE2-dA/dG and 4OHE2-A/G standards prepared from the quinone reaction with dA/dG, showing +78 × 1 and +78 × 2 Da products. The corresponding ATR-IR spectra of 4OHEE2, dA, and purified 4OHEE2-dA shown in the inset, verified the blocked N–H groups in 4OHEE2-dA. (B) The LC-MRM chromatogram of oxidative and 4OHE2-adducted lesions (8oxo/4OHE2-dG) acquired from the pellet (top) or supernatant (bottom) of the chromatin DNA treated with 30 μM 4OHEE2 (right) versus the control (left). No 4OHEE2-dA signals were detected. (C) The LC-MRM chromatogram of the released depurinating adducts acquired from the final postplating culture medium of the 4OHE2-treated MCF-7 cells (right) versus the control (left). *Retention time shifted by 2.0 min compared to the standard. Data acquired without (orange) and with (blue) IM-CHO microdroplet derivatization.

From the treated chromatin DNA (right of Figure 4B), 4OHEE2-dG and 8oxo-dG were detected from the pellet of hydrolyzate (top right of Figure 4B), and both signals were enhanced (>10 times) by IM-CHO derivatization. In contrast to the low derivatization ratio with the 4OHEE2-dG standard prepared from dG, the signal of 4OHEE2-dG obtained from chromatin DNA was significantly enhanced (>10 times) by IM-CHO derivatization without inducing side reactions such as depurination. The enhanced signal likely originates from nonreleased N7-4OHEE2-dG adducts that persist due to slow depurination kinetics, leaving the tagging site accessible. This supports the ability of microdroplet derivatization to preserve fragile N7-dG modifications. Alternatively, the 4OHEE2 adduction site in chromatin DNA may differ from that in the nucleoside standard because of microenvironmental effects, yielding additional nontagging sites. Both possibilities are consistent with the MS^2^ spectra previously obtained from treated chromatin DNA, although the fragment ions still do not definitively identify the adduction site. The depurinating adduct 4OHEE2-G and its derivatized form (4OHEE2-G-IM) were detected in the hydrolyzate supernatant, with the derivatized signal enhanced by more than 10-fold. Only trace amounts of 4OHEE2-A-IM were detectable, and only after derivatization. The minimal adenine adducts detected here are consistent with previous observations.^44^ No 4OHEE2-dG or 4OHEE2-G signal was observed in the chromatin-DNA control, confirming the absence of endogenous 4OHEE2 adducts in MCF-7 cells. An appreciable 8-oxo-dG-IM signal was detected in the control following IM-CHO derivatization, whereas the nonderivatized signal was barely detectable, indicating endogenous oxidized dG—but not dA—in MCF-7 cells. Overall, these results support a guanine-dominant damage pattern and align with the GC-rich transcription-associated domains mapped previously by Click-seq.^44^ The weak 4OHEE2-A-IM signal in treated chromatin DNA suggests that only trace levels of depurinating 4OHEE2-A are released, and these are detectable only with IM-CHO derivatization.

Depurinating adducts (4OHE2-G/A) released into the culture media were collected every 24 h from 4OHE2-treated MCF-7 cells. The culture media from the first to fourth treatments (1 μM 4OHE2 each) and the fifth treatment (30 μM 4OHE2) were prepared. As shown in Figure 4C, no depurinating adducts could be detected from the control medium (left), while significant depurinating 4OHE2 adducts (right) were detected from the medium of the final treated cells. In contrast to the undetected A adducts from chromatin DNA, depurinating adducts of not only N7-4OHE2-G but also N3-4OHE2-A were detected from the cell medium (right of Figure 4C), and both signals were significantly enhanced by IM-CHO derivatization. Since the depurinating adducts released into the cell medium originate from chromatin or mitochondrial DNA, these variations may indicate distinct patterns of damage between the two subcellular compartments. It is also likely due to much faster depurination kinetics from dA in chromatin, resulting in a considerable amount of released A-adducts in the cell medium. Compared to the chromatin DNA, the cell medium sample exhibited some additional minor enhanced signals. We suspect that different positional isomers of depurinating adducts may be present in the cell medium since no enhanced signal was observed from the control (left of Figure 4C). Notably, the primary 4OHE2-G signal detected from the cell medium showed a 2 min retention time shift, but its MS^2^ spectrum was essentially the same as that from the standard (see Figure S10).

Calibration curves for the depurinating adduct standards (N7-4OHE2/4OHEE2-G, N3-4OHE2/4OHEE2-A), oxidized purine standards (8-oxo-dG, 8-oxo-dA), and the stable 4OHEE2-dG standard (Figure 5), along with 4OHE1-G used as the IS^44^ (SI Figure S6B), were established using LC-MRM. Reversed-phase C18 separation showed excellent chromatographic resolution (Figure 5A), indicating that microdroplet derivatization did not alter retention behavior. The calibration curves displayed strong linearity (*R* ^2^ > 0.99) and demonstrated a 13–43-fold increase in sensitivity (slope enhancement) following IM-CHO microdroplet derivatization (Figure 5B). Method validation (Table S2) yielded limits of detection (LOD) and quantification (LOQ) of 0.05–28 fmol on-column for the IM-derivatized (+78) species, with accuracies >92% and intra/inter-day precision <13% across all analytes using both low and high QC standards. Matrix effects were evaluated by comparing 4OHE1-G-IM signals in water, cell-culture medium, and chromatin hydrolyzate, along with their respective negative controls (SI Figure S11). No detectable 4OHE1-G or 4OHE1-G-IM signal was observed in any zero-spike control, confirming the absence of background interference or matrix-induced ionization enhancement. Calibration curves generated in all three matrices showed good linearity (*R* ^2^ > 0.92) and similar enhancement factors upon IM-CHO derivatization. Although matrix suppression reduced the 4OHE1-G-IM signal—particularly in cell medium—IS effectively corrected for these matrix effects. For example (Figure S11B), quantification of 8-oxo-dG-IM (200 ppb) in cell-culture medium using water-based enhanced calibration curves and IS (4OHE1-G) normalization yielded an accurate value of 196 ppb (2% error), compared to 100 ppb (50% error) without IS correction. These results confirm that IS normalization fully compensates for matrix-dependent signal suppression. IS normalization effectively corrected these differences. Therefore, the enhanced calibration curves prepared in water, together with 4OHE1-G-IM (10 ppb) as the IS, were used to quantify depurinating adducts in cell medium and both oxidized and stable DNA adducts in chromatin DNA.

**Figure 5:**
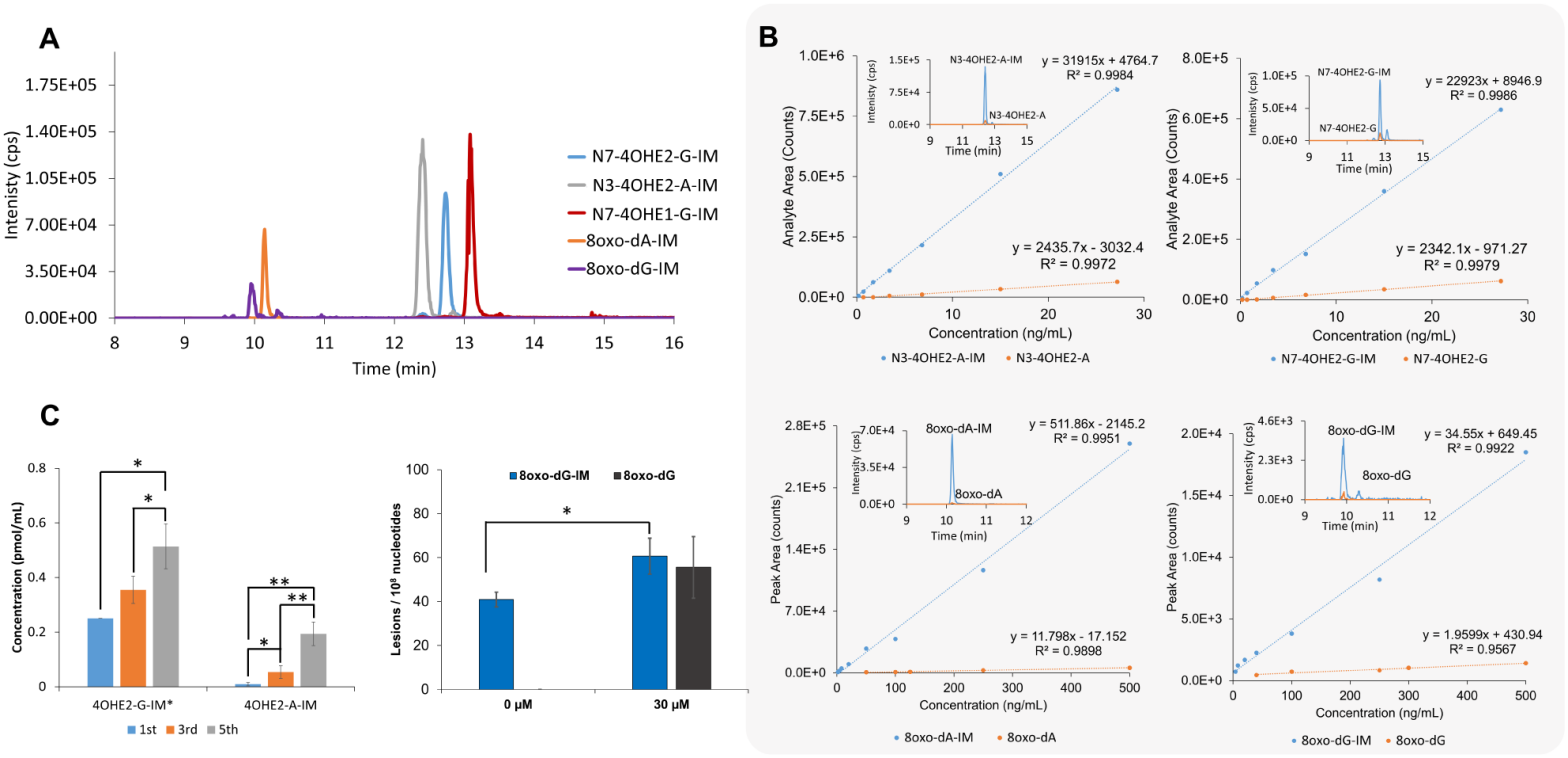
Microdroplet-induced IM-CHO online postcolumn derivatization for quantification of DNA damage caused by 4OHE2/4OHEE2 using LC-MRM. (A) LC chromatogram of all standards. (B) Calibration curves of N3-4OHE2-A, N7-4OHE2-G, and 8-oxodG/dA with (blue) and without (orange) IM-CHO derivatization, showing sensitivity enhancement (calibration slope) by 13–43 folds. The extracted ion chromatograms are shown in the inset, showing signal enhancement by >10 times. (C) Quantification results of oxidative lesions (Figure 4B) detected from chromatin DNA (right) and depurinating adducts (Figure 4C) released into the cell medium (left). Each value was expressed as the mean (*n* = 3) and standard deviation (±SD), *P < 0.05, **P < 0.01. Data acquired without (orange) and with (blue) IM-CHO derivatization.

The concentrations in cell media were converted to reflect corresponding concentrations in the cell growth medium (∼17 mL/dish). As summarized on the left side of Figure 5C, the levels of both N3-4OHE2-A and N7-4OHE2-G adducts released from the cells significantly (*p* < 0.05) increased from the first to the fifth posttreatment, indicating a time-dependent release. The concentration of 4OHE2-induced adducts from the culture media of MCF-10F cells was approximately 2–5 pmol/mL, quantified using LC-electrochemical detection.^50^ The concentration measured from MCF-7 cells was about ten times lower. However, the required sample amount (5 μL from one culture dish) was estimated to be merely 10% of that used in previous studies (25% of cells from 5 T-75 flasks). The concentration of 8oxo-dG detected from chromatin DNA was converted to the nucleotide damage rates based on the normalization of total nonmodified nucleobase concentrations (dA + dT + dG + dC), which were quantified using LC-UV detection^5,51^ (see Figure S12). The oxidative damage level was estimated at approximately 40 lesions per 10^8^ nucleotides in the control group and increased to about 60 lesions per 10^8^ nucleotides under treatment with 30 μM 4OHE2 (right side of Figure 5C). These damage rates fall within the reported range of 10–100 lesions per 10^8^ nucleotides seen in mouse tissue DNA.^52^ Additionally, chromatin DNA purified from single culture dish was also sufficient for detecting oxidative or adducted lesions.

We further verified the accuracy of our quantification by comparing results obtained with IM-CHO derivatization to those obtained without derivatization, as well as to previously reported values generated from independent cell batches. For side-by-side comparison, the oxidation level of dG (8oxo-dG-IM) in 4OHEE2 (30 μM)-treated chromatin DNA (Figure 5C, right) was determined to be 60 ± 8.15 lesions/10^8^ nucleotides (*n* = 3), which did not differ significantly from values obtained without derivatization. In the control sample (Figure 5C, left), the endogenous oxidation level was 40 ± 3.36 lesions/10^8^ nucleotides (*n* = 3) with IM-CHO derivatization, whereas the nonderivatized signal was too weak for reliable quantification (S/*N* < 3; Figure 4B). For batch-to-batch comparison, the levels of 4OHEE2-G-IM and 4OHEE2-dG-IM measured in 4OHEE2-treated chromatin DNA (Figure 4B) were ∼3 ± 1 (*n* = 3) and ∼3 ± 0.24 (*n* = 3) lesions/10^8^ nucleotides, respectively, consistent with previously reported values^44^ obtained from separate cell batches without derivatization (Figure S13). These consistent results reinforce that estrogen metabolite-induced covalent DNA lesions differ from the R-loop-associated damage generated through estrogen receptor-mediated transcriptional activity.^53^

## Conclusion

Compared to conventional derivatization techniques for modified NBs or nucleosides (Table 1), one of the primary benefits of this method is its ultrafast reaction with high yield and without artifacts. These features enable, to our knowledge, the only online postcolumn derivatization method for NBs or nucleosides (Table 1), with time savings of 4–5 orders of magnitude. This method reduces sample loss during the reaction and posttreatments, thereby reducing the required sample amount by an order of magnitude. Additionally, this method tags the exocyclic amine (−NH_2_) or pyrrolic nitrogen (−NH) of NBs or nucleosides, greatly enhancing the detection sensitivity while preserving their intact structures. Consequently, it applies to a broader range of nucleic acid modifications, especially for modifications on dG, for which derivatization has not been addressed by conventional techniques.

It is noted that some sensitivity enhancements provided by this method are lower than those achieved with conventional methods, such as epigenetic marks (like 5me-C or 5f-C). Part of this shortfall is due to the lack of posttreatments, such as cleanup procedures to remove excess reagents and unwanted matrix components, as well as enrichment steps—such as extraction—to concentrate the derivatized products. These operations improve detection sensitivity by reducing ion-suppression sources and increasing analyte concentration prior to LC–MS analysis. In our online derivatization and *in situ* detection system, these postderivatization cleanup and enrichment steps are intentionally omitted to enable rapid, high-throughput analysis. Thus, the overall sensitivity gained by derivatization may somehow diminish. Additionally, the IM-CHO microdroplet derivatization method does not tag modifications on exocyclic amines, which are common sites for many metabolites, including HNE-dG/dA and dA-4OHE2. Nevertheless, microdroplet IM-CHO tagging can be used as a rapid assessment tool to characterize unknown modification sites by determining whether these modifications interfere with tagging sites.

While microdroplet-accelerated reactions have been reported for other compound classes, and some limitations are associated with the reported method, our work addresses a distinct and unmet challenge: enabling ultrafast, mild condensation between IM-CHO and NBs, whose ring nitrogens are intrinsically weak nucleophiles due to tautomerism and resonance delocalization. Positively charged microdroplets enrich and orient IM-CHO and NBs at the air–water interface, enabling instantaneous, catalyst-free condensation with selectivity governed by microsecond-scale kinetic and thermodynamic factors that differ fundamentally from bulk chemistry. This approach also integrates directly with LC–MS^2^, the gold standard for global analysis of modified nucleic acids. Together, these features provide a clear conceptual advance and justify the significance of applying microdroplet chemistry to NB modifications.

## Supplementary Material



## Notes

Experiments for cellular sample preparation (**SI method**), MS2 of each nonderivatized and IM-CHO-derivatized standard pairs (**Figure S1**), MRM-XICs to compare the bulk versus microdroplet derivatization of standards (**Figure S2**), examination of potential artifacts induced by microdroplet IM-CHO derivatization of dG/dA (**Figure S3**), comparison of microdroplet IM-CHO condensation with and without 0.1% formic acid (**Figure S4**), the full scan spectra of 4OHE1-G (200 ppb) acquired without and with IM-CHO spray under negative mode using the previous LC-MS method (**Figure S5**), quantification using 4OHE1-G as the internal standard (**Figure S6**), reproducibility and carryover of the online IM-CHO derivatization (**Figure S7**), IM-CHO derivatization of 4OHEE2-A/G and 4OHE2-dA/dG (**Figure S8**), high-resolution spectra of dA and 4OHEE2-dA standards (**Figure S9**), MS2 of 4OHE2-G-IM acquired from the treated culture medium (**Figure S10**), correction for matrix effect associated with IM-CHO derivatization (**Figure S11**), determination of nonmodified nucleosides from pellet hydrolyzate by LC-UV (**Figure S12**), batch-to-batch comparison for the quantification results without and with IM-CHO derivatization for different batches of the chromatin sample treated with 30 μM 4OHEE2 (**Figure S13**), MRM parameters for each standard (**Table S1**), and Figures of merit and validation data of DNA adducts (**Table S2**) (PDF)
