# Supplementary material for: Ultrafast Postcolumn Microdroplet Derivatization of Nucleobases for Enhanced Online Detection, Characterization, and Quantification of Nucleic Acid Modifications Using LC-MS2
Source: Anal Chem. 2026 May 28;98(22):16127–39. doi: 10.1021/acs.analchem.6c00004 (PMC13261616; doi:10.1021/acs.analchem.6c00004)
Supplement: Supplementary file 1 [file ac6c00004_si_001.pdf]

Supporting Information

Ultrafast Post-Column Microdroplet Derivatization of Nucleobases for Enhanced Online Detection, Characterization, and Quantification of Nucleic Acid Modifications Using LC-MS^2

Quynh-Trang Do, Husam Kafeenah, Ching-Hua Huang, Shu-Hui Chen*

Department of Chemistry, National Cheng Kung University, No.1 College Road, Tainan, 701, Taiwan

*Corresponding author: shchen@mail.ncku.edu.tw

Table of contents

Page S2 Supplementary methods

Page S4 **Figure S1**. MS2 of each non-derivatized and IM-CHO-derivatized standard pairs

Page S12 **Figure S2.** MRM-XICs to compare the bulk versus microdroplet derivatization of standards

Page S12 **Figure S3.** Examination of potential artifacts induced by microdroplet IM-CHO derivatization of dG/dA

Page S13 **Figure S4.** Comparison of microdroplet IM-CHO condensation with and without 0.1 % FA

Page S14 **Figure S5.** The full scan spectra of 4OHE1-G (200ppb) acquired without and with IM-CHO spray under negative mode using the previous LC-MS method

Page S14 **Figure S6.** Quantification using 4OHE1-G as the internal standard

Page S15 **Figure S7.** Reproducibility and carryover of the online IM-CHO derivatization

Page S15 **Figure S8.** IM-CHO derivatization of 4OHEE2-A/G and 4OHE2-dA/dG.

Page S16 **Figure S9.** High resolution spectra of dA and 4OHEE2-dA standards

Page S16 **Figure S10.** MS2 of 4OHE2-G-IM acquired from the treated culture medium

Page S17 **Figure S11.** Correction for matrix effect associated with IM-CHO derivatization

Page S18 **Figure S12.** Determination of non-modified nucleosides from pellet hydrolysate by LC-UV

Page S19 **Figure S13.** Batch-to-batch comparison for the quantification results without and with IM-CHO derivatization for different batches of the chromatin sample treated with 30 µM 4OHEE2

Page S20 **Table S1**. MRM parameters for each standard

Page S21 **Table S2.** Figures of merit and validation data of DNA adducts

Supplementary materials

Buffer and solvents. MS-grade acetonitrile (ACN), dimethyl sulfoxide (DMSO), and methanol were purchased from Merck (Darmstadt, Germany). LC-grade formic acid (FA) and MS-grade 3-nitrobenzyl alcohol (NBA) were purchased from Thermo Fisher Scientific (Walthan, MA, USA). LC-grade Methanol (MeOH) was obtained from Duksan (Kyungki-Do, Korea). MS-grade Isopropanol (IPA) and Formic acid (FA) were obtained from Honeywell/Riedel-de Haen (Seelze, Germany). MS-grade Acetone, Tris hydrochloride(TRIS-HCl), and sodium hydroxide (NaOH) were purchased from J.T.Baker (Rador, PA). Magnesium chloride, and manganese dioxide (#217646) were from Sigma-Aldrich (St. Louis, MO, USA).

Reagents for cell culture and chromatin extraction. Complete protease inhibitor cocktail (#11836170001) was purchased from Roche Life Sciences (Indianapolis, IN, USA). Micrococcal nuclease (#M0247S) was obtained from NEB. Trypsin-EDTA 0.5% (#15400054), minimum essential medium (MEM) (, and fetal bovine serum (FBS) were purchased from Invitrogen (Gibco, Gaithersburg). Antibiotic–antimycotic 100X (#CC501-0100) was obtained from genedirex (Taiwan).

Proteinase K (#P2308), nuclease P1 from penicillium citrinum (NP1) (#N8630), deoxyribonuclease I from bovine pancreas type II (DNase I) (#D4527), phosphatase alkaline from porcine kidney (ALP) (#1701302300), phosphodiesterase I from crotalus adamanteus venom (PDE I) (#P3243), dithiothreitol (DTT) (#D0632), paraformaldehyde (#158127), 4-(2-hydroxyethyl)piperazine-1-ethanesulfonic acid (HEPES), potassium chloride (KCl), phenol (#8.22296), chloroform, isoamyl alcohol, and bis(2-hydroxyethyl)amino-tris(hydroxymethyl)methane (BIS-TRIS) (#B9754) were purchased from Sigma-Aldrich (St. Louis, MO, USA). Polyethylene glycol tert-octylphenyl ether (Triton X-100) and glycine were purchased from JT Baker (Center Valley, PA).

Supplementary methods

Chromatin sample preparation. One dish of MCF-7 cells (1×10^7) were harvested and crosslinked with 1% formaldehyde. The reaction was quenched in 125 mM glycine. Cells were then re-suspended in 800 µL cytosol extraction buffer (10 mM HEPES, 10 mM KCl, 1.5 mM DTT, 1% Triton X-100, 1x protease inhibitor, 5% glycerol) and incubated on ice for 15 minutes to permeabilize the cells. The pellets were subsequently digested with 4000 gel units of MNase for 1 hour at 37 °C in 100 µL of digestion buffer (50 mM Tris-HCl [pH 7.9], 5 mM $CaCl_2$, 100 µl/mL bovine serum albumin) and arrested with 5 mM EDTA. The chromatin was harvested from the resulting supernatant by centrifugation at 16,000xg for 10 minutes and was determined to contain 500 µg total protein (determined by BCA assay) and 100 µg total DNA (measured by Thermo Scientific µdrop plate). The chromatin solution was incubated with the MnO_2-activated 4OHEE2 quinone (300 µM) at 37°C for 24 hr. Proteins and DNA were precipitated using 2 volumes of ACN containing 20 mM NaCl. The supernatant, containing depurinating adducts, was enriched by ACN salting-out extraction,[42] dried, re-suspended in 30 µL of H2O/ACN (80:20), and stored at -20°C until use. A volume of 5 µL was injected into microLC-MS.

The pellet containing stable adducts (4OHEE2-dG) was dissolved in PBS buffer, followed by Proteinase K (10 mg/mL) digestion, DNA extraction by phenol:chloroform:isoamyl alcohol (25:24:1) and precipitation with ice-cooled isopropanol. A tetra-enzyme mixture was used to hydrolyze DNA following previously reported method.[40] The stable 4OHEE2 adducts were enriched by ACN salting-out extraction,[42] dried, re-suspended in 30 µL of H2O/ACN (80:20) and stored at -20°C until use and 5 µL was injected for analysis.

Culture medium sample preparation. When cells reached 80% confluence, three dishes of culture media were changed to DMEM medium supplemented with 5% FBS and 1 µM 4OHE2 in DMSO. The media were collected after every 24-hour exposure to 4OHE2, counted as one-time treatment. The cells were then placed in new dishes and allowed to reach 80 % confluence for 48 hours in DMEM supplemented with 5% FBS prior to the next treatment with 1 µM 4OHE2. This process was continued for three treatments, followed by an increase in dosage to 30 µM 4OHE2 for the final treatment. The media collected at the 1st (24 hr postplating), 3rd (168 hr postplating) and 5th post-treatment (312 hr postplating) were supplemented with 2 mM ascorbic acid (Sigma-Aldrich) to prevent possible decomposition or oxidation of the compounds. The media from MCF-7 cells treated with DMSO were used as the control. The cell culture media (~50 mL in total) was loaded on a Sep-Pak C18 Vac Cartridge (500 mg, 6cc) obtained from Waters Ltd (Watford, UK), washed with 2 column volumes of 0.1 M potassium phosphate buffer (pH 7.5), and then eluted with 6 mL of elution buffer comprised of methanol: acetonitrile: water: formic acid (8:1:1:0.1). The solvent was evaporated under nitrogen flow and the residue was re-suspended in 500 µL of phosphate buffer (pH 7.5) and enriched by ACN salting-out extraction.[42] The organic layer containing DNA adducts were then collected, dried, and re-suspended in 160 µL of 20% acetonitrile in water and 5 µL was injected for analysis.

Total non-modified nucleobase quantification. The HPLC setup was formed of ECOM ECP2010 HPLC Pump, Thermo Dionex Ultimate 3000 VWD. Data acquisition, and processing were all carried out using chromeleon software (Thermo Scientific). Hydrolyzed DNA was diluted 4 times prior to inject onto a C18 column (150 x 4.6 mm , 5 µm , 90 A°, Varian). The detection wavelength and column temperature were set at 260 nm and 50 °C, respectively. The mobile phases were water with 10 mM ammonium formate (A) and methanol (B). The flow rate was 1 mL/min with starting condition of 5% B, which was held for 2 min, followed by a linear gradient to 30% B over 1 min, to 70% B over 1 min. The flow was held at 70% B for 4 min followed by re-equilibration for an additional 7 min. Under this condition, the non-modified nucleobases were eluted with a retention time 2.6 min (dC), 4.5 min (dG), 5.1 min (dT), and 5.8 min (dA). The quantification of non-modified nucleobases was quantified by the peak area based on freshly prepared calibration (**Figure S12**).

Figure S1. MS2 of each non-derivatized (top) and IM-derivatized (bottom) pairs of **A.** dC, **B.** 5me-dC, **C.** 5f-dC, **D.** dT, **E.** dA, **F.** rU, **G.** dG, **H.** 8oxo-dA, **I.** 8oxo-dG, **J.** N1me-dG, **K.** N2dime-dG, **L.** 4OHE1-G, **M.** 4OHE2-A, **N.** 4OHE2-G, **O.** 4OHE2-dG, **P.** 4OHE2-dA, **Q.** 4OHEE2-G, **R.** 4OHEE2-A, **S.** 4OHEE2-dG, **T.** 4OHEE2-dA. The transition ion pairs were circled in red and other cleavages were circled in blue.

Figure S1A

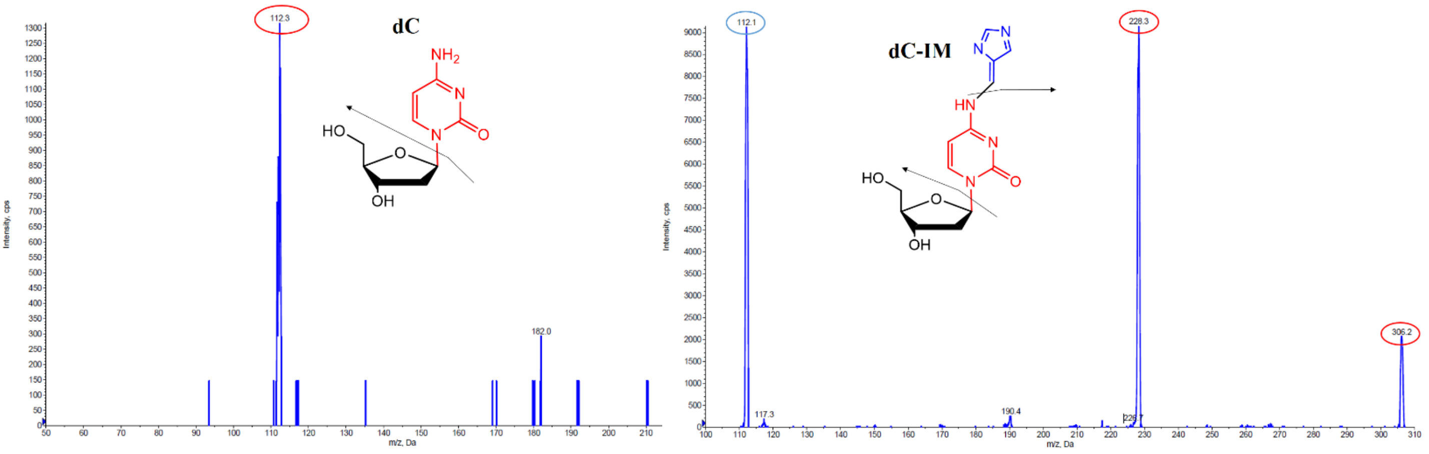

Figure S1B

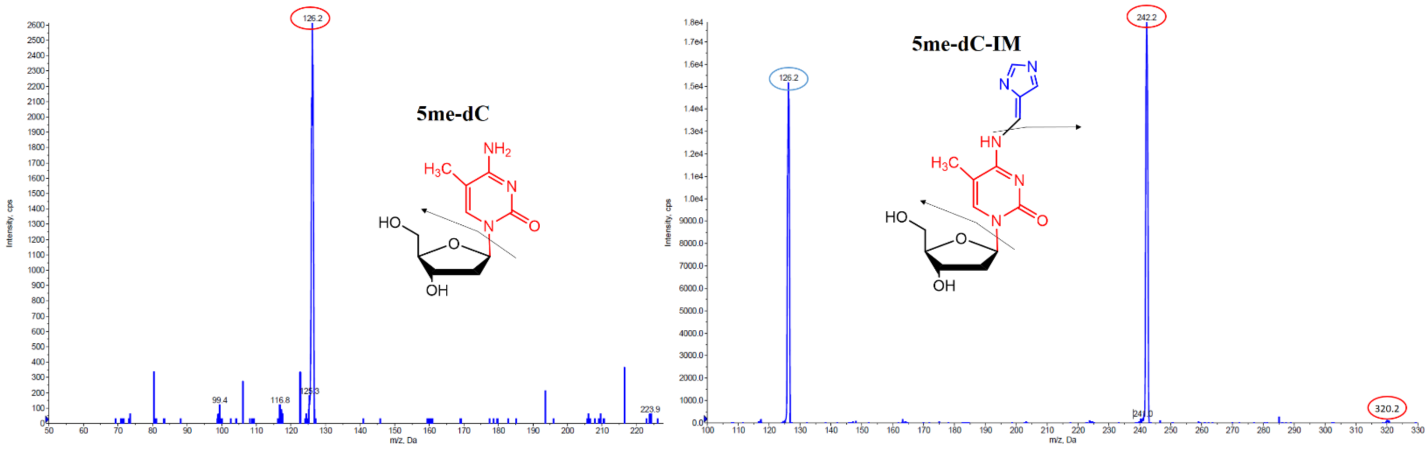

Figure S1C

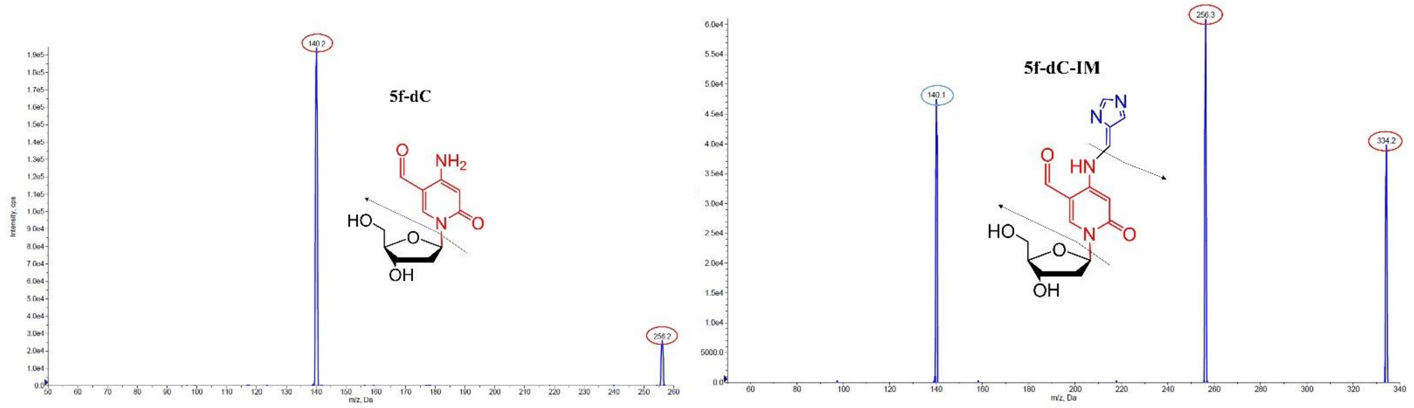

Figure S1D

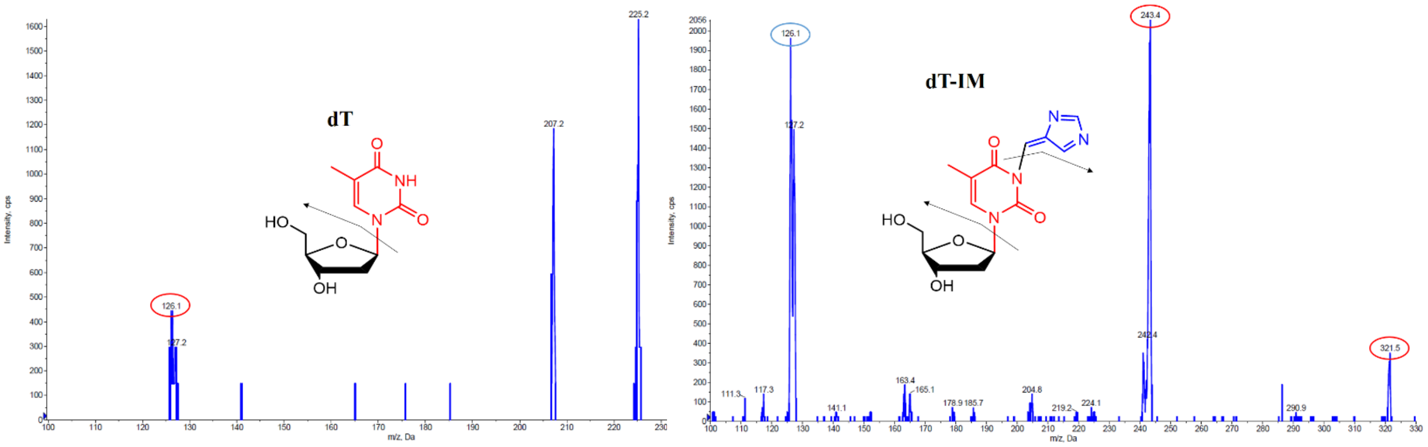

Figure S1E

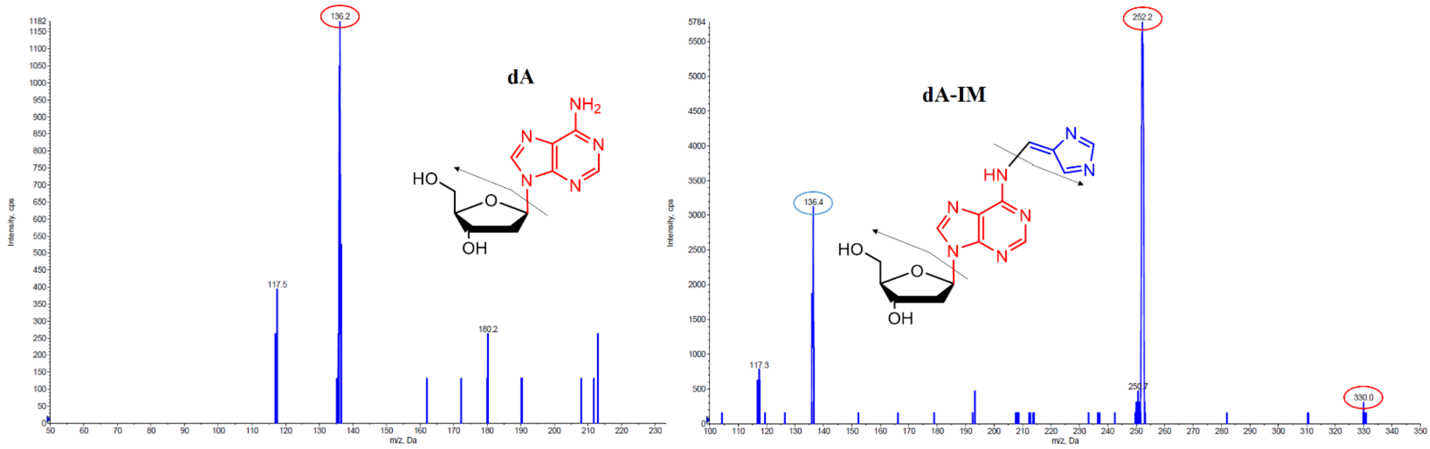

Figure S1F

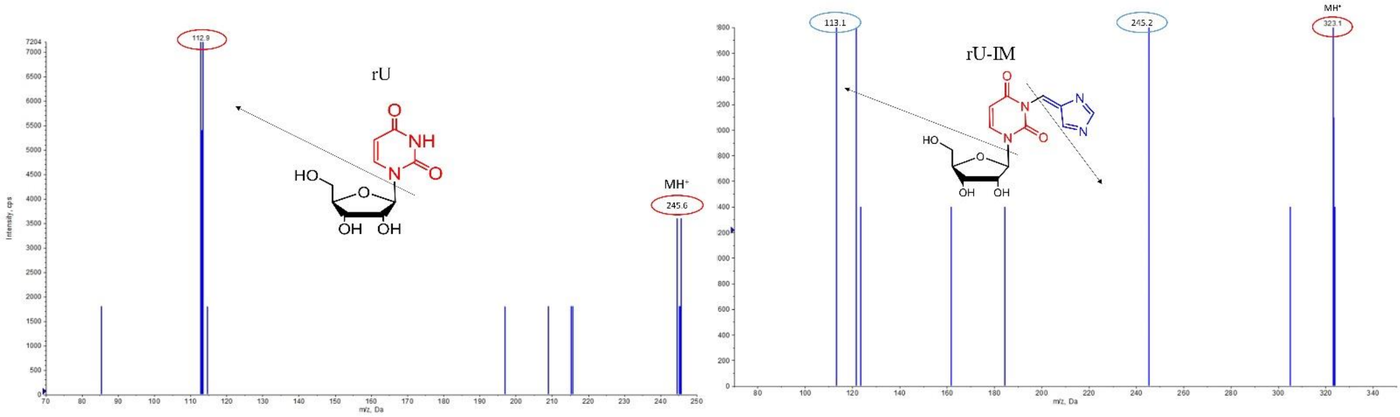

Figure S1G

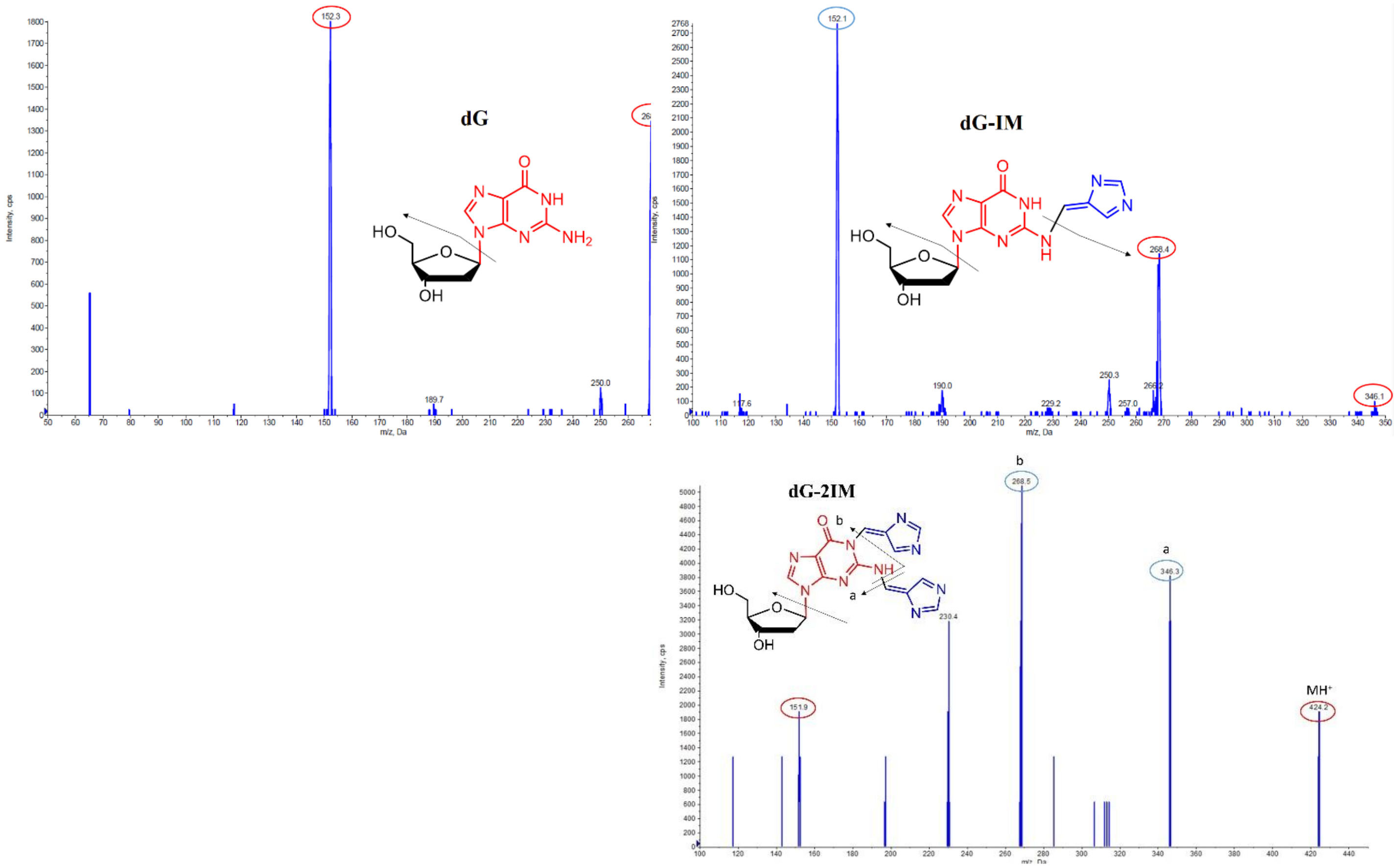

Figure S1H

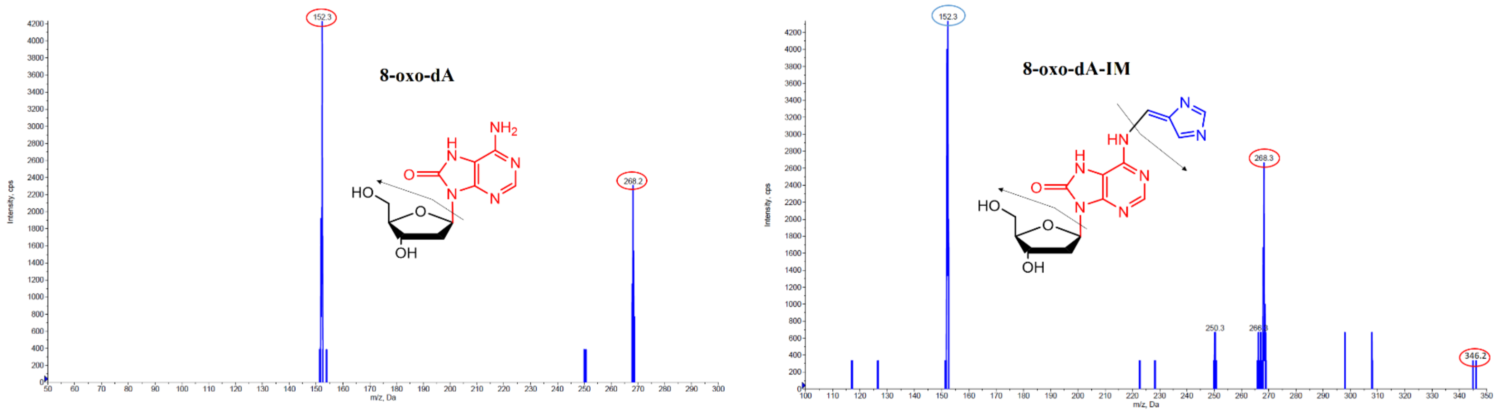

Figure S1I

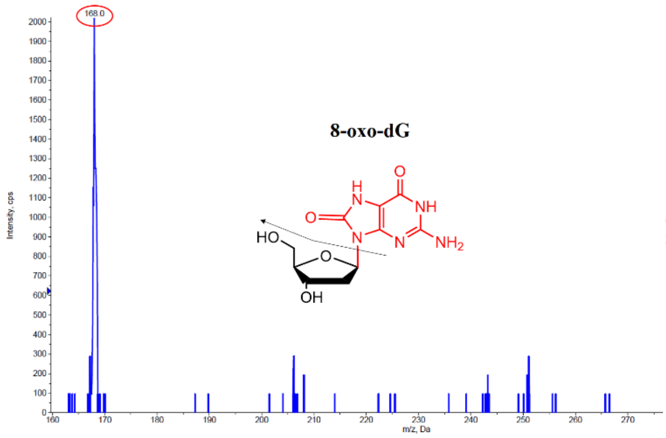

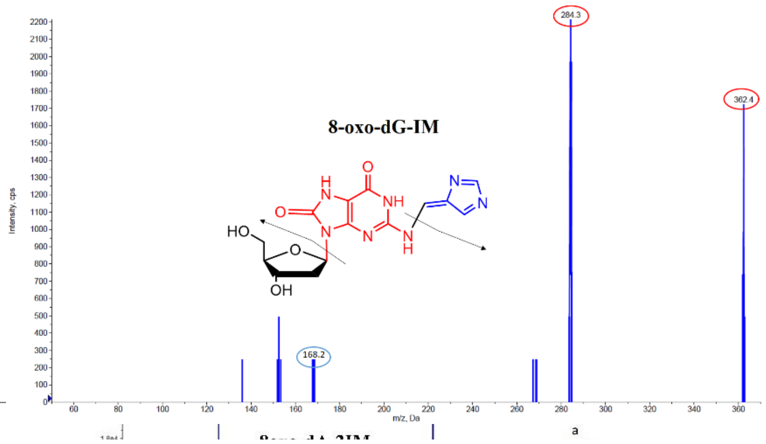

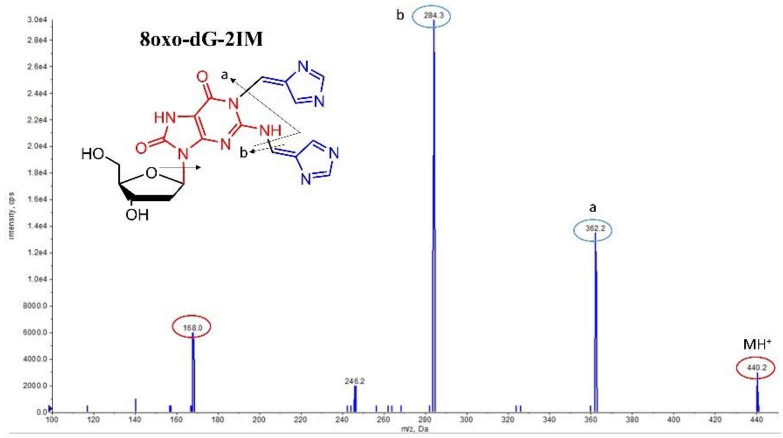

Figure S1J

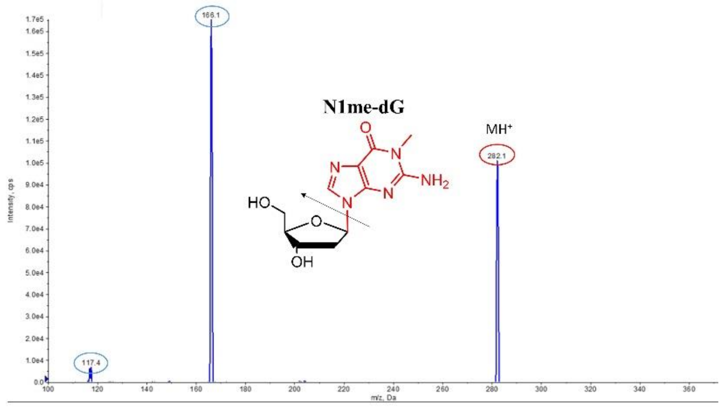

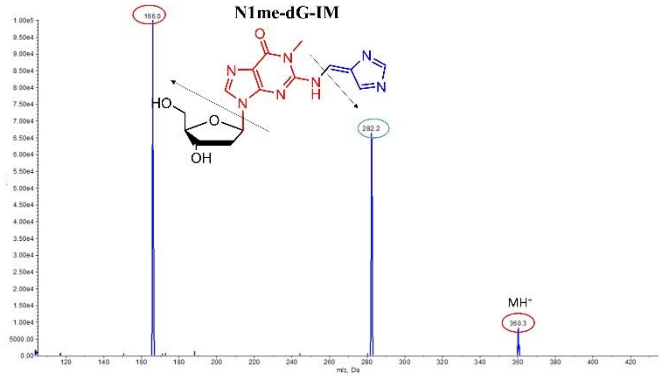

FigureS1K

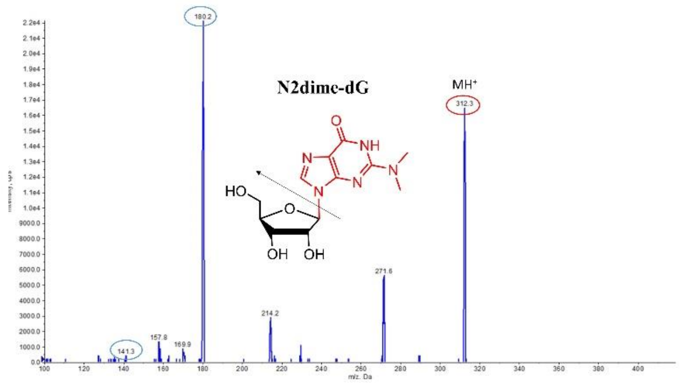

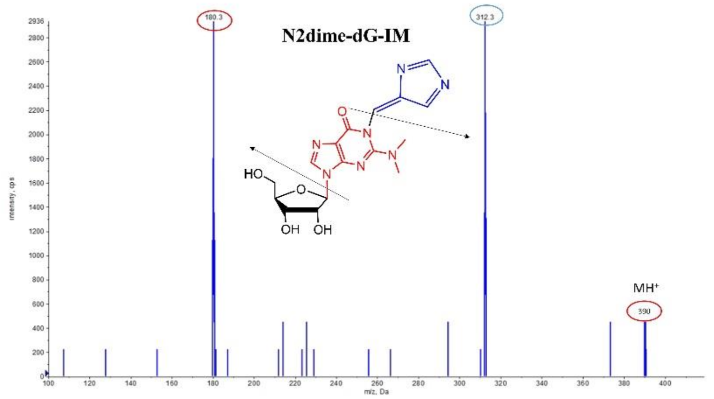

FigureS1L

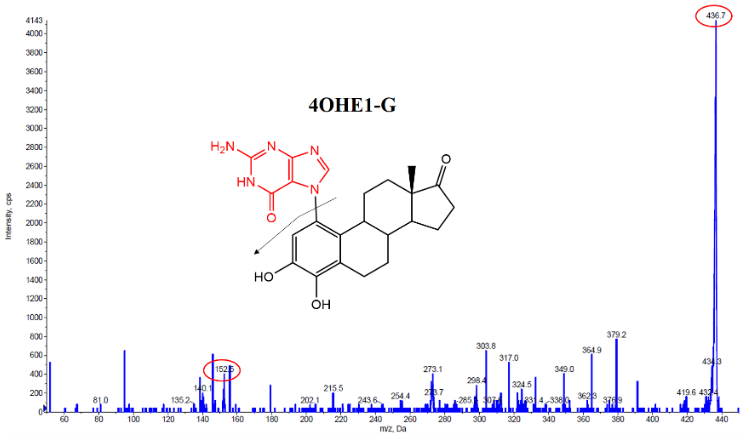

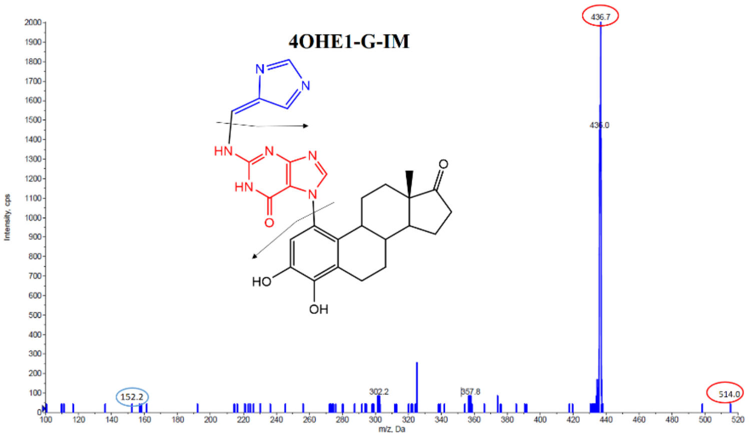

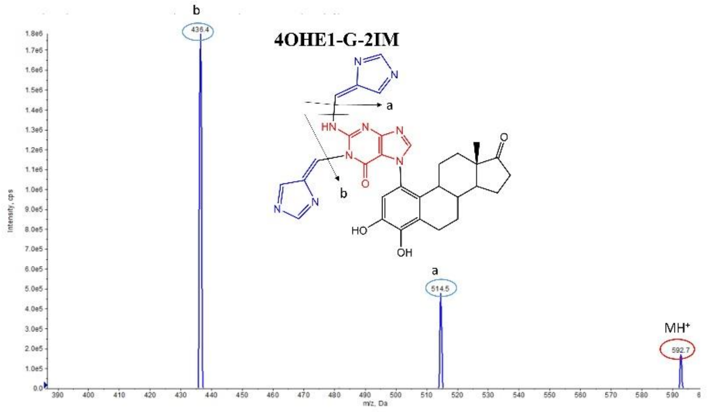

Figure S1M

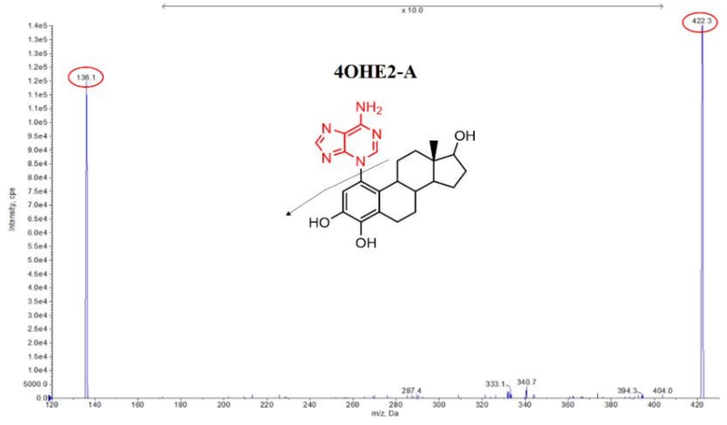

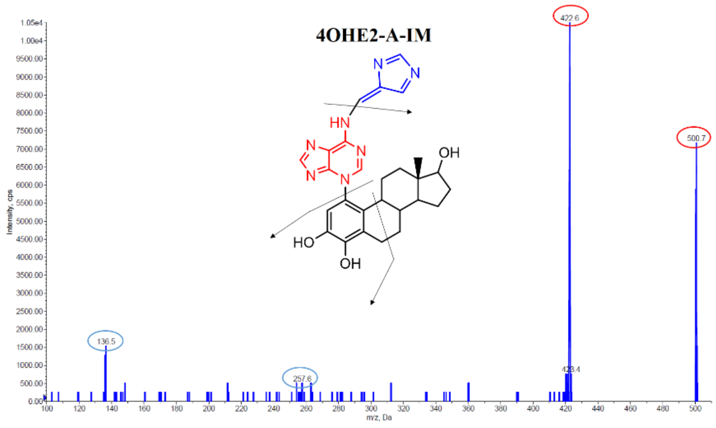

Figure S1N

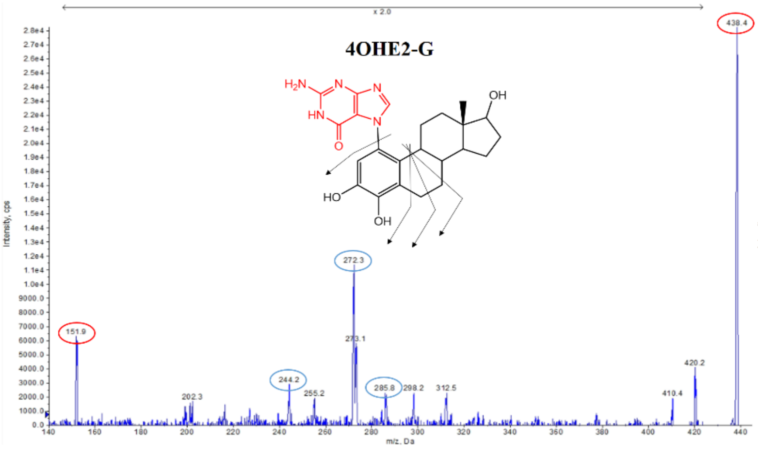

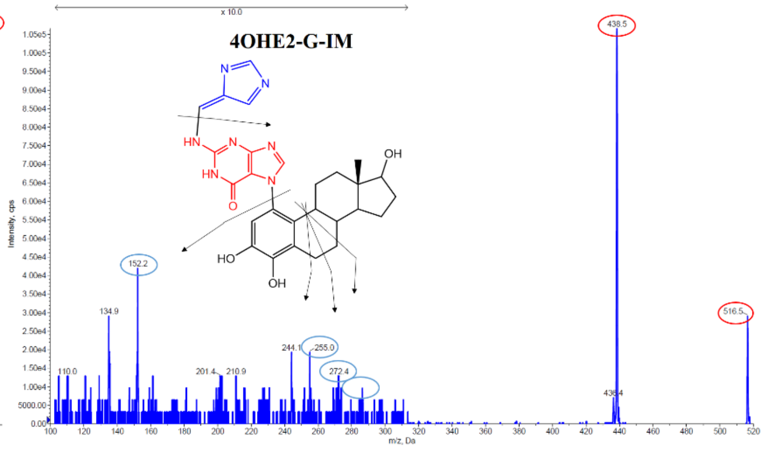

Figure S1O

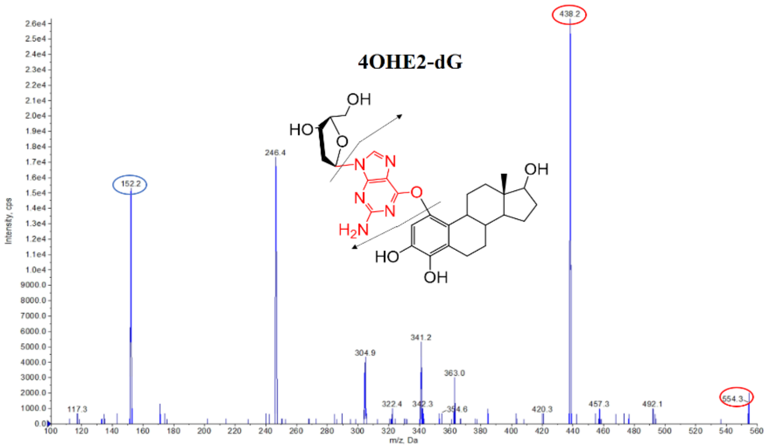

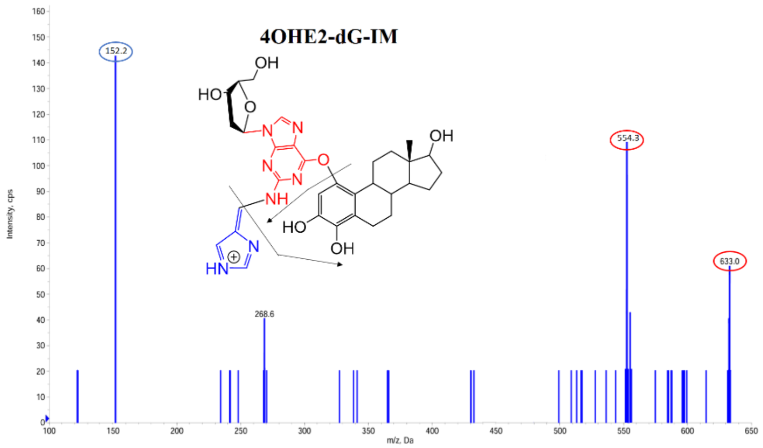

Figure S1P

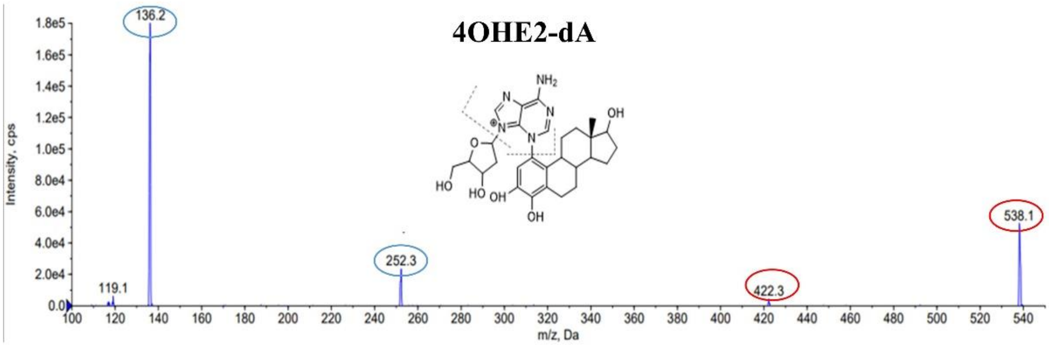

Figure S1Q

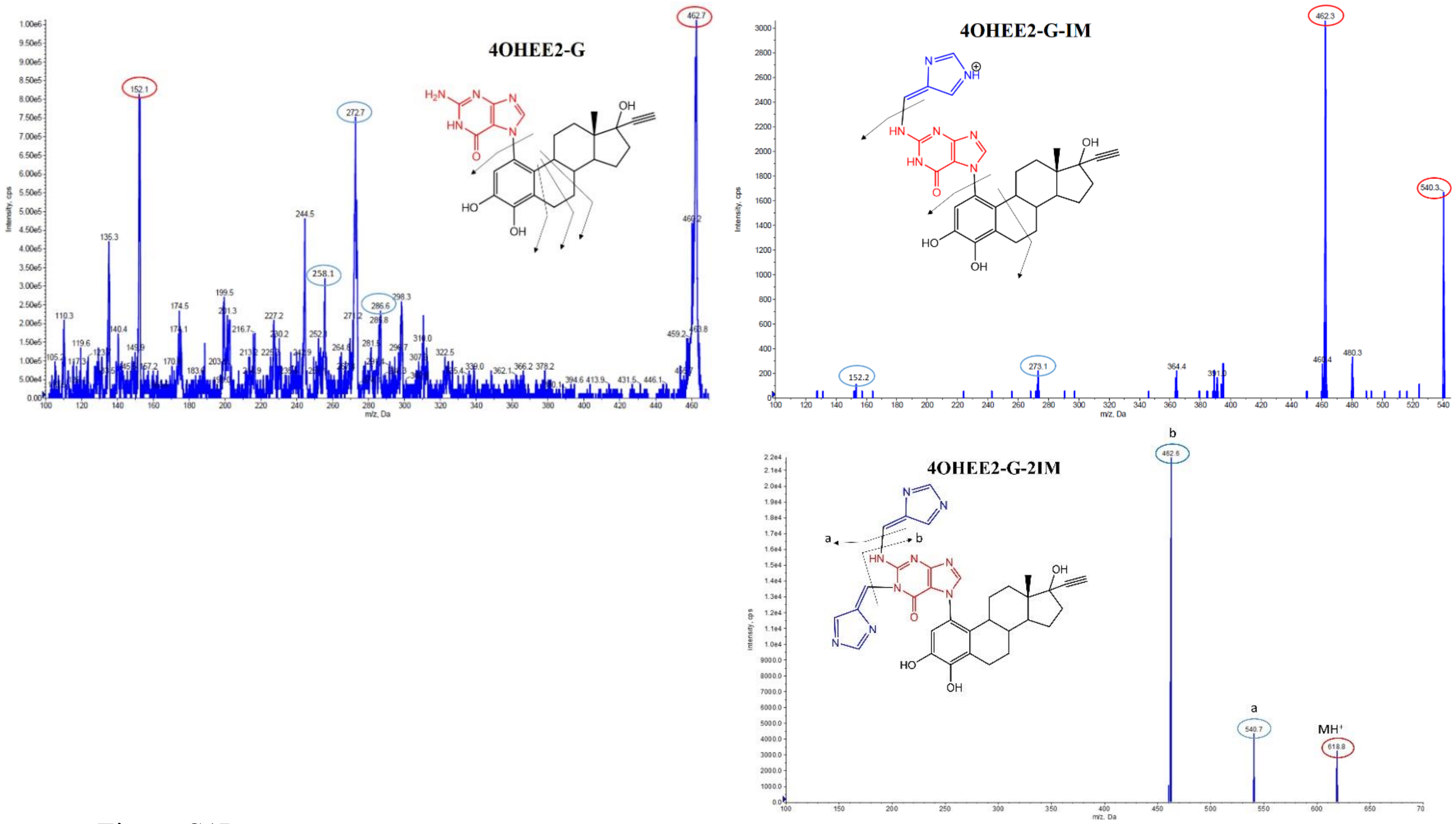

Figure S1R

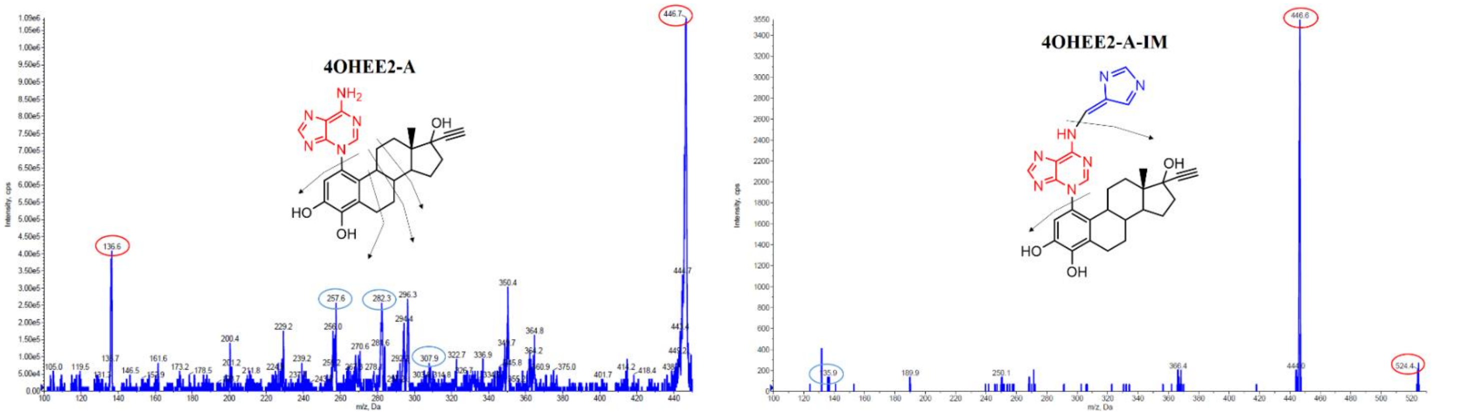

Figure S1S

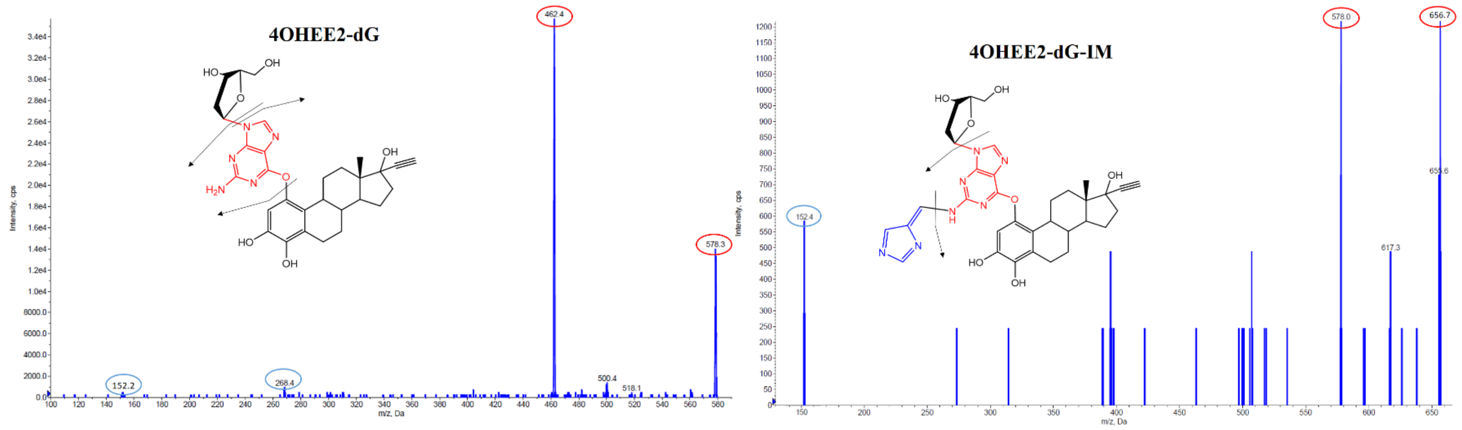

Figure S1T

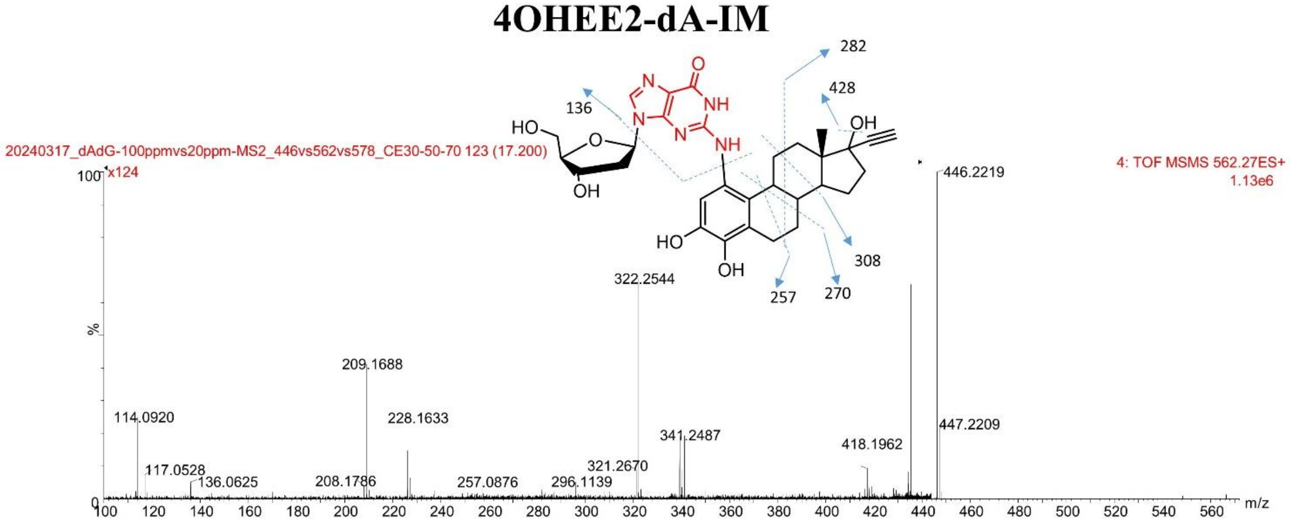
4OHEE2-dA-IM
282
428
136
HO
O
N
N
O
NH
N
N
NH
OH
OH
HO
OH
308
257
270
20240317_dAdG-100ppmvs20ppm-MS2_446vs562vs578_CE30-50-70 123 (17.200)
x124
4: TOF MSMS 562.27ES+
1.13e6
100
%
0
446.2219
322.2544
209.1688
114.0920
228.1633
341.2487
447.2209
418.1962
321.2670
117.0528
136.0625
208.1786
257.0876
296.1139
100 120 140 160 180 200 220 240 260 280 300 320 340 360 380 400 420 440 460 480 500 520 540 560
m/z

Figure S2. MRM-XICs to compare the bulk versus microdroplet derivatization of standards. Extracted ion chromatogram of each standard (0.2 µg/mL each, orange trace) and their corresponding IM-derivatized products (blue trace) acquired from the bulk reaction with IM (2 µg/mL in 0.1% FA) incubated at 80°C for 6hrs followed by LC-MS analysis versus online post-column microdroplet reaction. Each peak was enlarged in the inset.

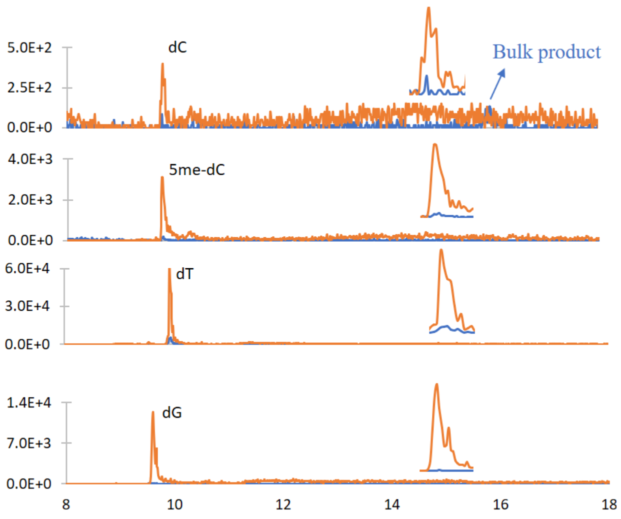

Figure S3. Examination of potential artifacts induced by microdroplet IM derivatization of dG/dA. MRM-XIC of **(A)** dG (top) and 8-oxo-dG (bottom), and **(B)** dA (top) and 8-oxo-dA (bottom). Blue indicates IM-derivatized compounds, and orange indicates compounds without IM derivatization.

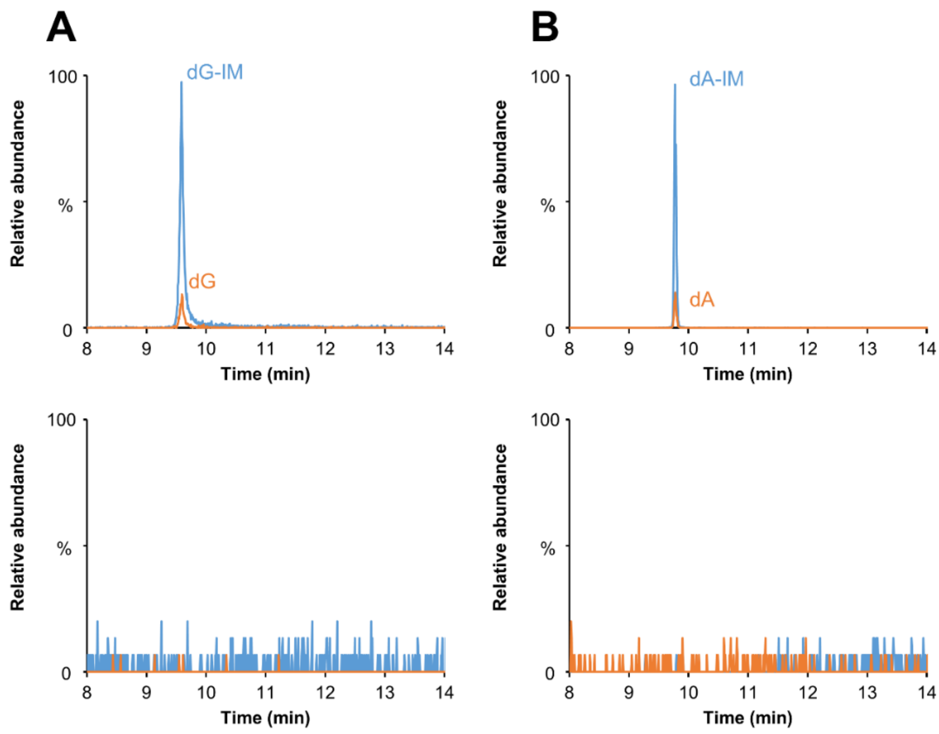

Figure S4. Comparison of microdroplet IM-CHO condensation with and without 0.1 % formic acid. MS¹ full-scan spectra of standard nucleobases (dA, dT, dC, dG) analyzed by online microdroplet fusion show stronger formation of IM-derivatized products (dA-IM, dT-IM, dC-IM, dG-IM, and dG-2IM) in the presence of 0.1% formic acid (top) compared to the reaction without acid (bottom).

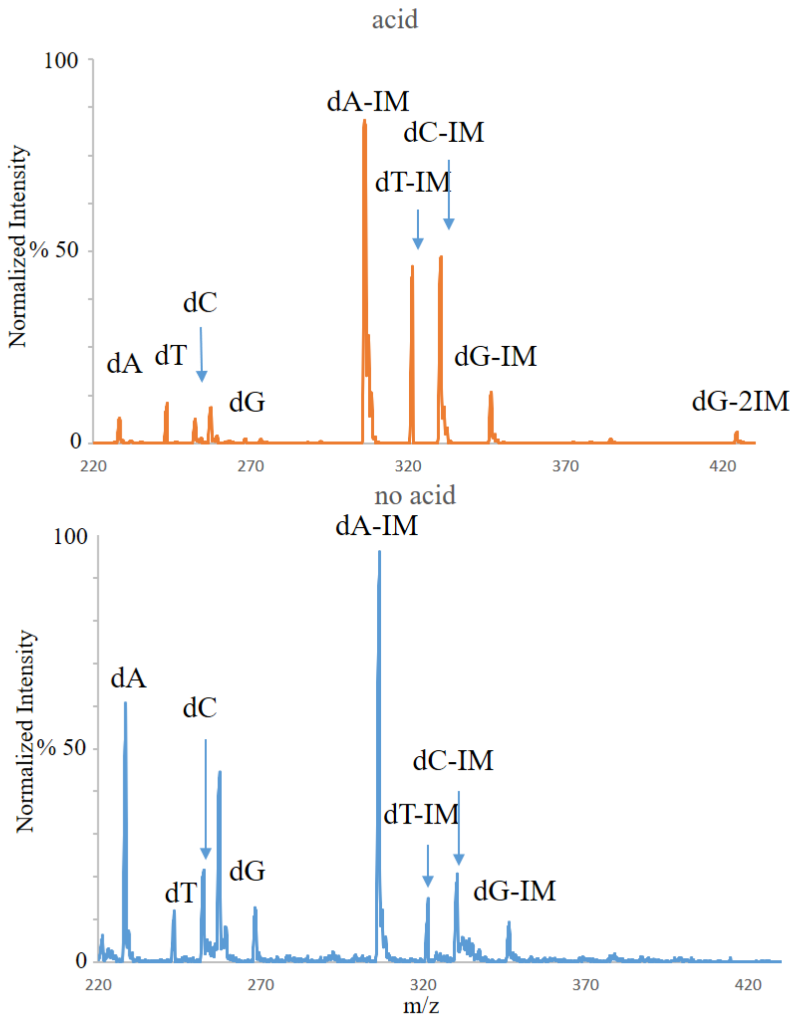

Figure S5. The full scan spectra of 4OHE1-G (200ppb) acquired without (left) and with (right) IM-CHO spray under negative mode using the previous LC-MS method.[44] The intensity of 4OHE1-G of the intact 4OHE1–G [M–H]⁻ ion (m/z = 434.6) remained unchanged or slightly increased, with no detectable imine or carbinolamine products (m/z =512.6).

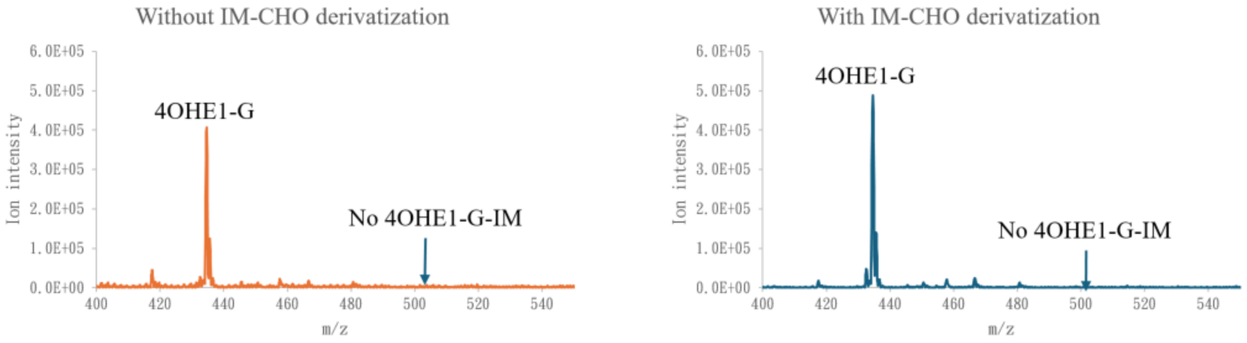

Figure S6. Quantification using 4OHE1-G as the internal standard. (A) Limit of quantification (LOQ) and (B) calibration curve of 4OHE1-G-IM at 10 pg/mL

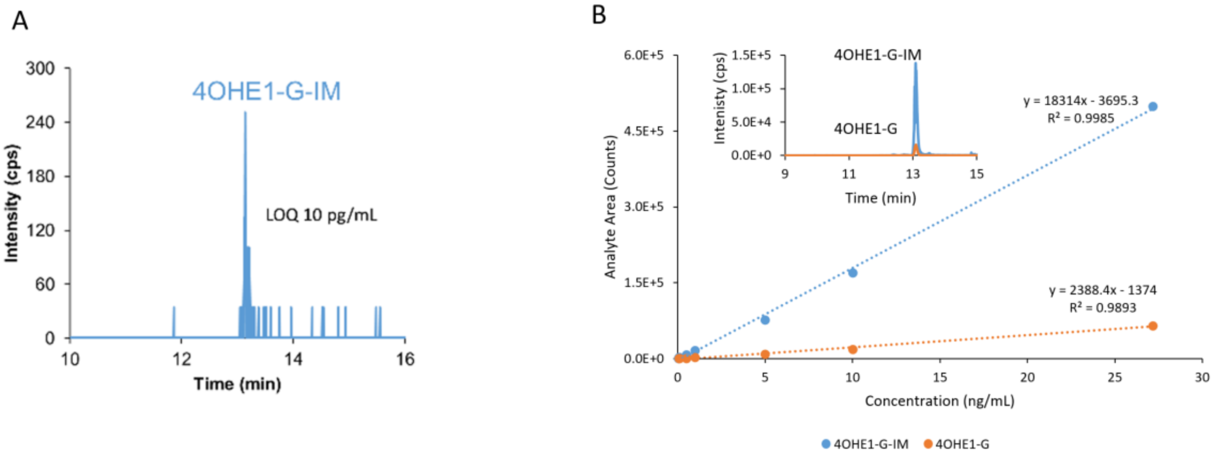

Figure S7. Reproducibility and carryover of the online IM-CHO derivatization. (A) Three consecutive injections (5uL) of 8oxo-dA (200ppb) or 4OHE1-G (200ppb); each injection followed by two blank injections for wash. *background noise present in both the sample and wash injection (B) IM-CHO contamination in instrument after overnight run and after 5 or 10 blank injections with continuous solvent (10%DMSO) wash spray.

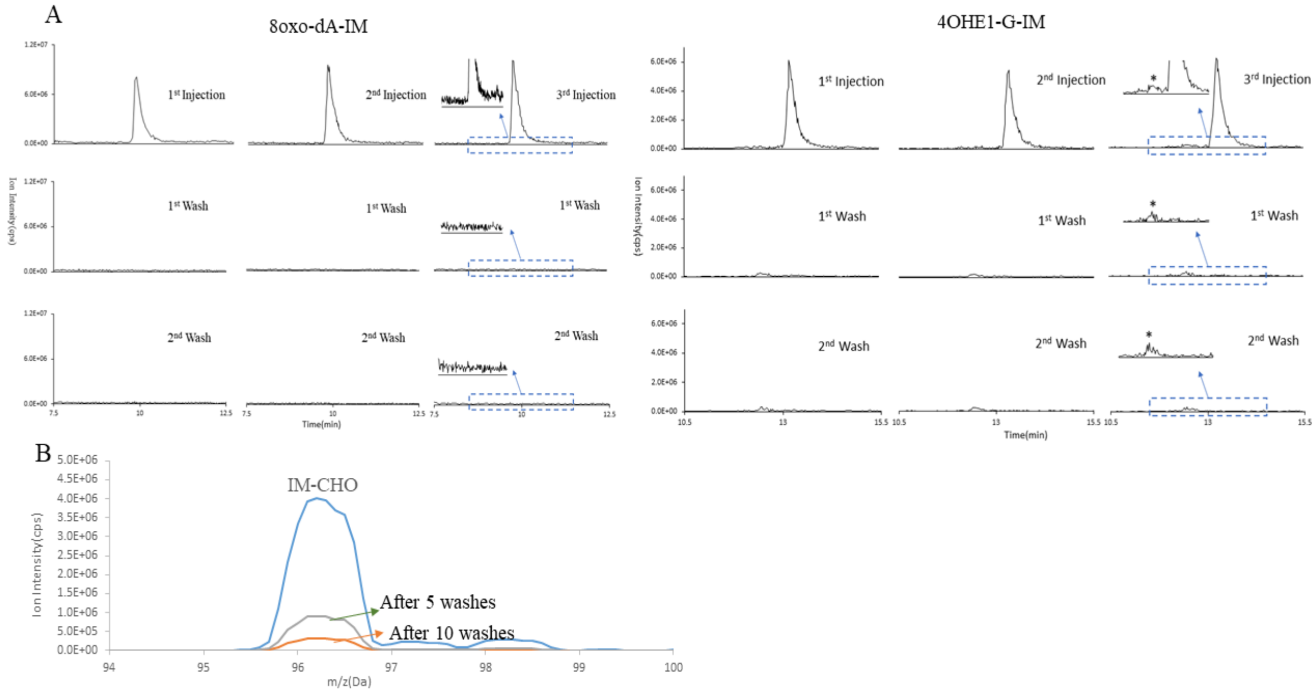

Figure S8. IM-CHO derivatization of 4OHEE2-A/G and 4OHE2-dA/dG.

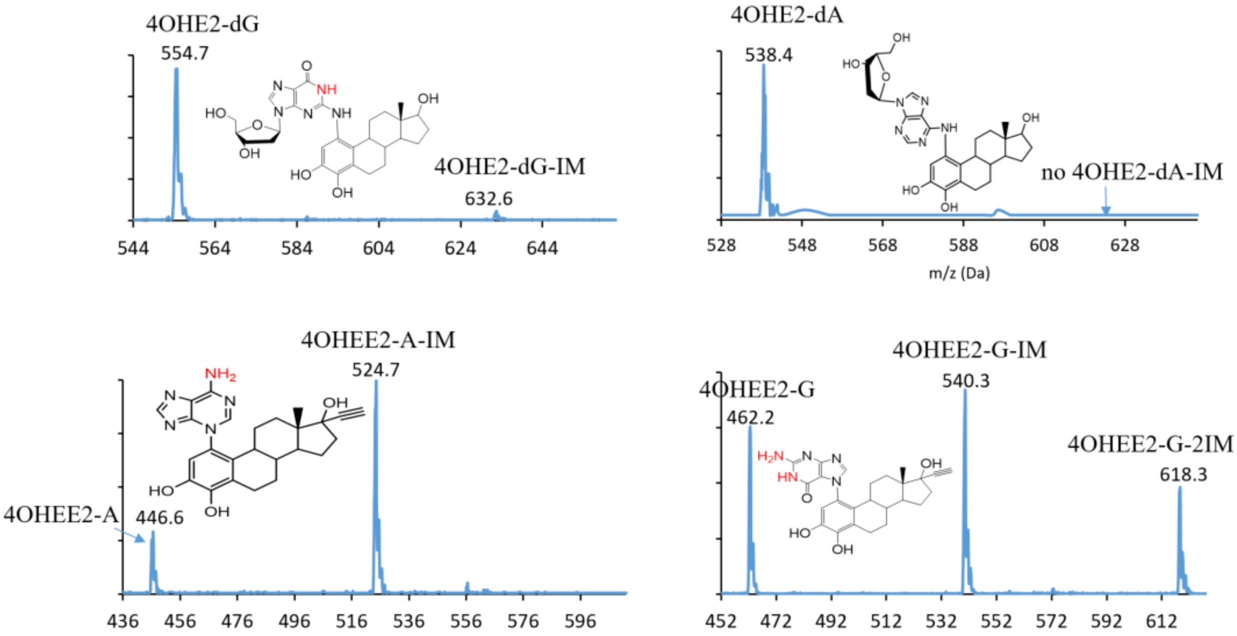

Figure S9. High resolution spectra of dA and 4OHEE2-dA standards. The mass accuracy is confirmed by comparing its accurate mass and isotopic pattern the theoretical value and pattern.

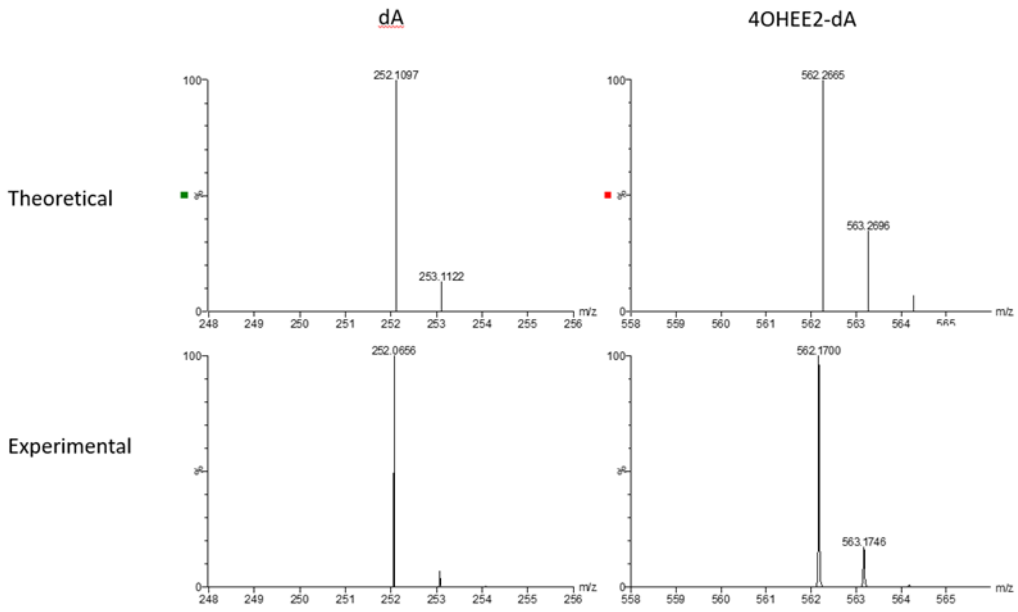

Figure S10. MS2 of 4OHE2-G-IM standard (top) and 4OHE2-G-IM* (2-min shift in retention time compared to the standard) acquired from the treated culture medium (bottom)

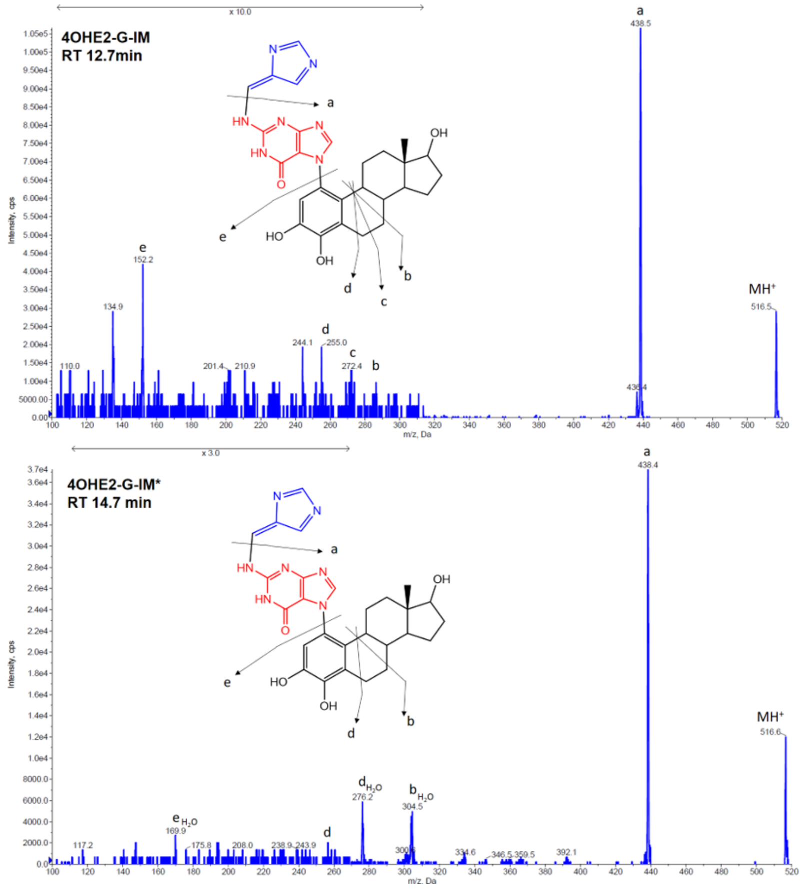

Figure S11. Correction for matrix effect associated with IM-CHO derivatization. (A) The signal of the derivatized 4OHE1-G-IM (100 ppb) in water, chromatin DNA, and cell medium along with their corresponding controls. No background signal was enhanced and greater matrix suppression was observed from cell medium. (B) Calibration curves of 8oxo-dG-IM established in water without (orange) and with (green) 4OHE1-G (10 ppb) as the IS. 8oxo-dG (200 ppb) in cell medium was determined to be ~100 ppb (50% error) without IS and ~196 ppb (2% error) with IS from the respective regression lines.

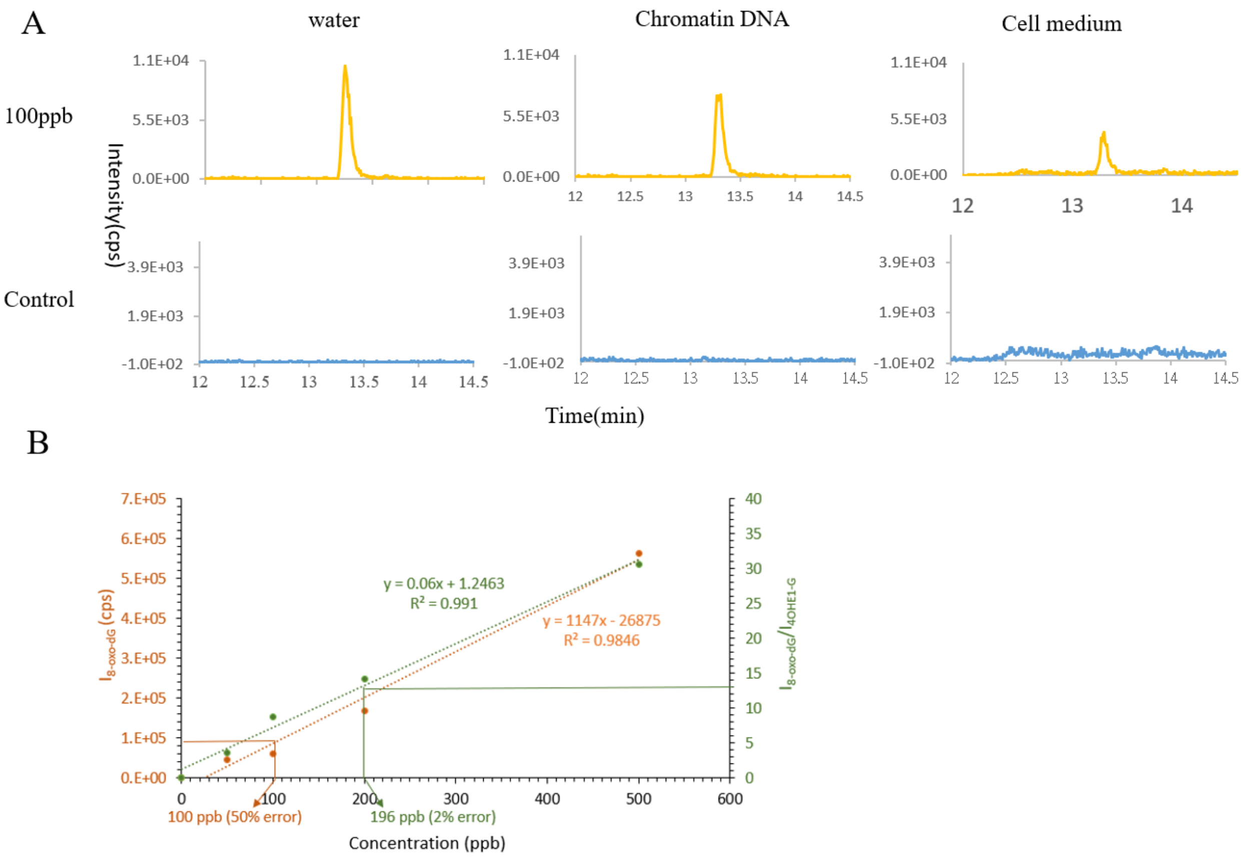

Figure S12. Determination of non-modified nucleosides from pellet hydrolysate by LC-UV. (A) Chromatogram of a standards mixture (left), hydrolyzed DNA from chromatin treated with solvent (middle), and hydrolyzed DNA from chromatin treated with 4OHEE2 (right). Calibration curves of non-modified nucleobases are shown in panels (B), (C), (D), and (E). The calculated amounts of individual nucleosides in the hydrolyzed DNA samples from chromatin treated with solvent (control) and with 4OHEE2 (experiment) are also presented in (F).

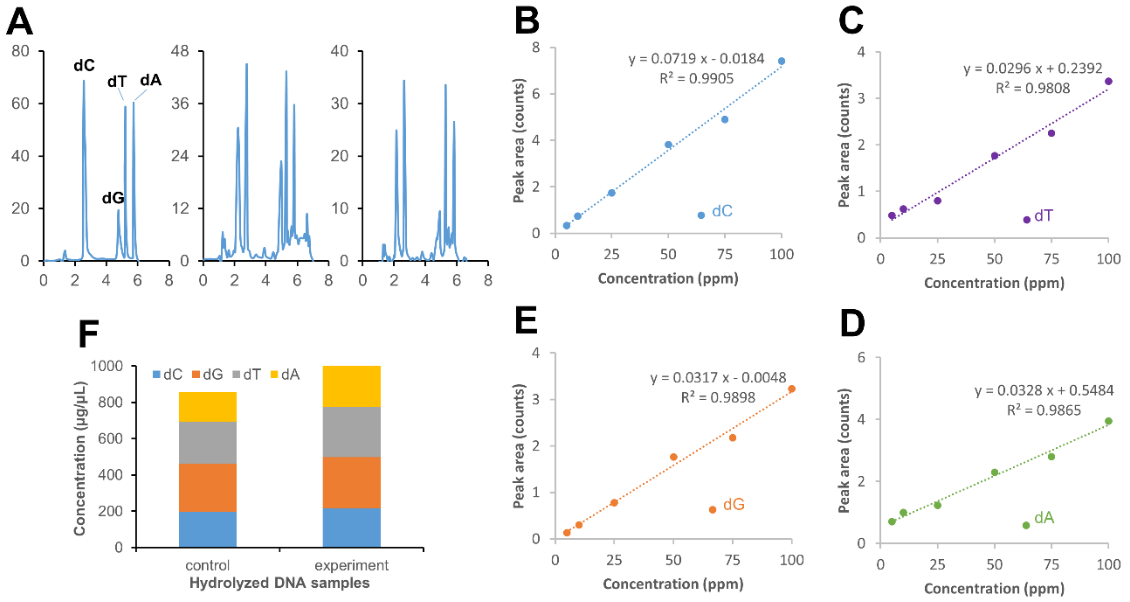

Figure S13. Batch-to-batch comparison for the quantification results without and with IM-CHO derivatization for different batches of the chromatin sample treated with 30 μM 4OHEE2. The damage or adduction levels in total DNA obtained (4OHEE2-dG-IM and 4OHEE2-G-IM) from this study (Figure 4B) using IM-CHO derivatization were compared with the previously reported values (4OHEE2-dG and 4OHEE2-G shown Figure 1 of reference 44) without derivatization.[44] No significance difference ($p<0.05$) was noted. Each value was expressed as the mean (n =3) and standard deviation (±SD).

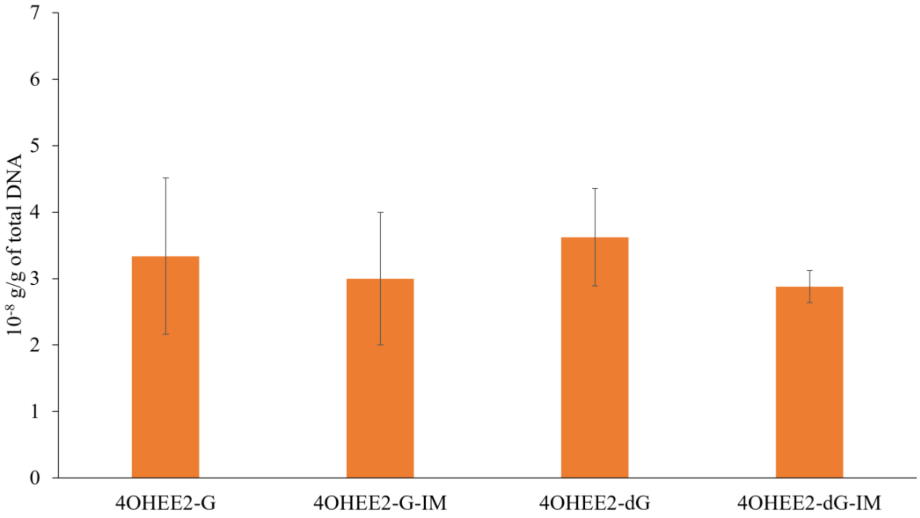

Table S1. MRM parameters for each standard.

Compound	Precursor $[M-H]^+$ (Da)	Product ion (Da)	Collision energy (V)	DP (V)	EP (V)	CXP (V)
dA	252	136	19	70	9	13
dA-IM	330	252	15	40	8	8
dG	268	152	15	70	7	12
dG-IM	346	268	7	40	8	14
dC	228	112	20	70	10	13
dC-IM	306	228	9	40	10	12
dT	243	127	13	70	7	11
dT-IM	321	243	11	40	5	7
5me-dC	242	126	20	70	10	9
5me-dC-IM	320	242	12	40	11	7
8-oxo-dG	284	168	10	50	7	11
8-oxo-dG-IM	362	284	10	50	9	10
8-oxo-dA	268	152	10	50	7	11
8-oxo-dA-IM	346	268	10	50	9	10
5-fdC	256	140	18	50	10	15
5-fdC-IM	334	256	15	50	10	15
4OHE1-G	436	152	62	188	9	24
4OHE1-G-IM	514	436	20	50	10	15
4OHEE2-A	446	136	62	188	9	24
4OHEE2-A-IM	524	446	20	50	10	15
4OHEE2-G	462	152	62	188	9	24
4OHEE2-G-IM	540	462	20	50	10	15
4OHEE2-dG	578	462	23	135	7	18
4OHEE2-dG-IM	656	578	15	50	10	15
4OHE2-A	422	136	62	188	9	24
4OHE2-A-IM	500	422	20	50	10	15
4OHE2-G	438	152	62	188	9	24
4OHE2-G-IM	516	438	20	50	10	15
4OHE2-dG	554	438	34	86	7	15
4OHE2-dG-IM	632	554	14	50	9	11

Table S2. Figures of merit and validation data of DNA adducts

Compound name	LOD (pg/mL)	LOQ (pg/mL)	On-column		Retention time (min)	Accuracy % (n=3)		Within-day precision % (n=5)		Between – day precision % (n=3)		Calibration range (pg/mL)	R^2
			LOD (fmol)	LOQ (fmol)		Low	Hi	Low	Hi	Low	Hi		
4OHE1-G-IM	5	10	0.05	0.10	13.11	97	96	5	7	7	6	10 – 27200	0.9983
4OHEE2-G-IM	25	50	0.23	0.46	13.33	95	98	11	9	8	9	170 - 27200	0.9967
4OHEE2-A-IM	25	50	0.24	0.48	12.98	104	97	8	12	12	5	170 - 27200	0.9929
4OHE2-G-IM	25	87	0.24	0.48	12.74	97	97	9	7	11	8	170 - 27200	0.9986
4OHE2-A-IM	25	87	0.25	0.5	12.40	97	96	10	9	10	3	170 – 27200	0.9984
8-oxo-dG-IM	1000	2000	14	27	10.3	101	107	12	6	7	6	2000 - 500000	0.9951
8-oxo-dA-IM	2000	4000	28	56	10.5	92	103	10	5	13	3	4000 - 500000	0.9922
